# An αvβ6/αvβ8-targeting peptibody inhibits integrin-dependent TGFβ activation, enables tumor-selective cytotoxic delivery, and synergizes with PD-L1 immune checkpoint blockade

**DOI:** 10.7150/thno.131237

**Published:** 2026-07-29

**Authors:** Angelo Corti, Anna Maria Gasparri, Barbara Colombo, Giulia Taiè, Elisa Sangiovanni, Leopoldo Sitia, Valeria Barreca, Alice Schirru, Alessandro Gori, Luc Garrigues, Sophie Chabot, Annapaola Andolfo, Maurilio Ponzoni, Anna Mondino, Arianna Pocaterra, Claudio Doglioni, Flavio Curnis

**Affiliations:** 1 Tumor Biology and Vascular Targeting Unit, Comprehensive Cancer Center, IRCCS San Raffaele Scientific Institute, Milan, Italy.; 2 Lymphocyte Activation Unit, Division of Immunology, IRCCS San Raffaele Scientific Institute, Milan, Italy.; 3 Istituto di Scienze e Tecnologie Chimiche (SCITEC-CNR), National Research Council of Italy, Milan, Italy.; 4 Translational Biology, In vivo Pharmacology and Analytical Chemistry, Evotec, 195, Route D’Espagne, 31100, Toulouse, France.; 5 Proteomics and Metabolomics Facility, Center for Omics Sciences, IRCCS San Raffaele, Milan, Italy.; 6 Pathology Unit, IRCCS San Raffaele Scientific Institute, Milan, Italy.; 7 Vita-Salute San Raffaele University, Milan, Italy.

**Keywords:** cancer, chromogranin A, TGFβ, peptibody, αvβ6 and αvβ8 integrins

## Abstract

**Background:**

The αvβ6 and αvβ8 integrins are upregulated in many solid-tumors and drive local activation of transforming growth factor-β (TGFβ), promoting immune evasion and resistance to immune checkpoint blockade. The chromogranin A-derived peptide **4Δ** targets the RGD-binding site of αvβ6/αvβ8 and inhibits integrin-dependent TGFβ-activation. We investigated whether **4Δ**-derived peptibodies can deliver cytotoxic drugs to cancer cells and enhance immune checkpoint inhibitors (ICIs) activity.

**Methods:**

Peptide **4Δ** was genetically fused to the Fc domains of murine and human IgG1 to generate the peptibodies **4Δ**mFc and **4Δ**hFc. Integrin-binding properties and inhibition of TGFβ activation were characterized using biochemical and cell-based assays. Peptibody internalization and lysosomal trafficking were analyzed by live-cell/confocal microscopy. Cytotoxic activity of peptibodies complexed with anti-Fc antibodies coupled to anticancer drugs was evaluated using αvβ6/αvβ8-positive and -negative cancer cells. Pharmacokinetics and antitumor activity of **4Δ**mFc, alone or in combination with an anti–PD-L1 antibody, were evaluated in murine fibrosarcoma and mammary carcinoma models.

**Results:**

**4Δ**mFc and **4Δ**hFc bound αvβ6 and αvβ8 with sub-nanomolar affinity. Both compounds selectively recognized αvβ6/αvβ8-positive tumor cells, as well as human pancreatic, lung, and colon carcinomas sections. **4Δ**mFc and **4Δ**hFc efficiently inhibited TGFβ activation, underwent efficient internalization, and trafficked to lysosomes. **4Δ**hFc enabled delivery of cytotoxic payloads to αvβ6/αvβ8-positive cells, including MMAE, MMAF, DM1, PBD, or DX8951. **4Δ**mFc delayed the growth of fibrosarcomas and improved mice survival in the mammary adenocarcinoma model when combined with an anti-PD-L1 mAb (immune checkpoint inhibitor), without overt toxicity.

**Conclusion:**

These peptibodies represent a dual-selective αvβ6/αvβ8-targeting platform that couples potent blockade of integrin-dependent TGFβ activation with efficient, selective delivery of cytotoxic payloads to cancer cells. These properties, together with the observed synergism with anti-PD-L1 mAbs, suggest their potential use as ligands for delivering cytotoxic agents to tumors, while concomitantly modulating the TGFβ-driven immunosuppressive microenvironment, either alone or in combination with ICIs.

## Introduction

Integrins, a family of cell surface receptors with a role in cell adhesion and signaling, have emerged as promising targets for cancer therapy. Among them, αvβ6 and αvβ8 integrins have been implicated in the immune escape of cancer cells through the activation and release of TGFβ, an immunosuppressive cytokine that promotes tumor growth and inhibits anti-tumor immune responses 1-7.

The integrin αvβ6 is overexpressed by head and neck squamous cell carcinoma, pancreatic ductal adenocarcinoma, liver, colon, ovarian, and breast cancers, and others 8-14, with important prognostic implications 8, 11, 15-17, while αvβ8 is overexpressed by various carcinoma cells and by tumor-infiltrating regulatory T cells (T_regs_) 6, 7, 18. Thus, in principle, targeting these integrins with compounds selective for their active site could potentially disrupt the immunosuppressive microenvironment of the tumor and enhance the efficacy of cytotoxic anti-cancer drugs and immunotherapies. According to this view, studies in animal models have shown that both anti-αvβ6 and anti-αvβ8 antibodies can induce significant antitumor responses through TGFβ-regulated mechanisms 4, 7, 19. This suggests that bi-specific compounds targeting the RGD binding site of both integrins may represent important inhibitors of TGFβ-mediated immunosuppressive mechanisms.

In this regard, we have previously described a chromogranin A-derived peptide (FETLRGDLRILSILRX_1_QNLX_2_KELQD, with X_1_ and X_2_ forming a triazole bridge) capable of recognizing the RGD-binding site of both αvβ6 and αvβ8 with high affinity and selectivity 20, 21. This peptide, called “peptide **5a**”, contains the canonical RGDLXXL αvβ6-integrin recognition motif followed by an amphipathic α-helix chemically stabilized by the triazole bridge 21, 22. This peptide efficiently accumulates on αvβ6- or αvβ8-positive tumors 20, 21 and, upon chemical conjugation to human serum albumin, can block TGFβ activation in various *in vitro* and *in vivo* tumor models 23.

The present study was undertaken to explore whether a related chromogranin A–derived peptide (FETLRGDLRILSILRHQNLLKEL; hereafter referred to as **4Δ**) could be exploited to develop a novel multifunctional platform capable of inhibiting TGFβ activation in tumors, delivering cytotoxic payloads to cancer cells, and enhancing the efficacy of immunotherapies. To this aim and to have at hand a homogeneous multivalent compound with a prolonged circulating half-life, we have prepared a peptide-antibody fusion product (called *peptibody*) consisting of a peptide **4Δ** fused to the Fc domain of murine or human IgG1 (**4Δ**mFc and **4Δ**hFc, respectively). The rationale for this choice relies on the fact that peptibodies can be produced by recombinant DNA technologies, thereby avoiding the need for chemical conjugation reactions typically yielding heterogeneous products, as occurred with the** 5a**-albumin conjugate. We show that these peptibodies recognize αvβ6^+^ and/or αvβ8^+^ cancer cells (either when cultured *in vitro* or in human cancer tissue sections), inhibit TGFβ activation, and reduce tumor growth in murine models. Furthermore, we demonstrate that these compounds can be used for delivering cytotoxic drugs or imaging agents to cancer cells, as well as for enhancing the antitumor efficacy of an immune checkpoint inhibitor (an anti-PD-L1 antibody) in murine models. These findings establish our peptibodies as a novel theranostic platform capable of both delivering drugs or imaging agents to tumors and simultaneously attenuating TGFβ-dependent immunosuppressive mechanisms within the tumor microenvironment.

## Materials and Methods

### Cell lines

TS/A murine mammary adenocarcinoma cells were from Sigma-Aldrich (cat. SCC177). 5637 human bladder carcinoma (cat. HTB-9) cells, LN-229 human glioblastoma (cat. CRL-2611) cells, WEHI-164 murine fibrosarcoma (cat. CRL-1751) cells, and BxPC-3 human pancreatic ductal adenocarcinoma (PDAC) (cat. CRL-1687) cells were purchased from ATCC.

T3M-4 human PDAC cells were obtained from Dr. M. Casucci (Milan). β6 and β8 integrin double knockout T3M-4 cells were generated via the CRISPR-Cas9 system using custom sets of 3 guide RNAs per gene (for human ITGB6: 5’-CCAGCUCCUUUAUUGUGUUG-3’, 5’-UGAGGUAAUACAAAUCCACC-3’, and 5’-AGGUGGUGCGCAGACUCUGC-3’; for human ITGB8: 5’-UUCUCCCGUGACUUUCGUCU-3’, 5’-UCCACAGGAUAUUUCUUCAG, and 5’-AUAGAAAAAUUAAAUUCCGU-3’; Labospace, Italy). Edited cells (ITGB6/B8-KO) were used as a bulk polyclonal pool without subcloning. BxPC-3, 5637, TS/A, T3M-4, and T3M-4 ITGB6/B8KO cells were cultured in RPMI-1640 (Euroclone, cat. ECB9006L) containing standard supplements and heat-inactivated fetal bovine serum (FBS, 10% v/v). LN-229 and WEHI-164 cells were cultured in DMEM (Euroclone, Cat. ECB7501L) containing standard supplements and 10% FBS. All cell cultures were periodically tested for mycoplasma contamination using the MycoplasmaCheck service provided by Eurofins Genomics. Cell surface integrin (αv, β1-subunit, α5β1, α8β1, αvβ3, αvβ5, αvβ6, and αvβ8) and PD-L1 expression were analyzed by flow cytometry as described previously 21, 22.

### Antibodies, integrins, and reagents

The following mouse anti-human integrin monoclonal antibodies were purchased from Merck: anti-αvβ6 mAb 10D5 (IgG2a, cat. MAB2077Z), anti-αvβ5 mAb P1F6 (IgG1, cat. MAB1961), anti-αvβ3 LM609 (IgG1, cat. MAB1976), anti-αv subunit mAb P3G8 (IgG1, cat. MAB1953Z), anti-β1 subunit mAb P5D2 (IgG2b, cat. MAB1959Z) and control IgG isotype mAb MOPC-31C (IgG1, cat. M9035). Mouse anti-human α8-subunit mAb 411709 (IgG1) was from R&D Systems (cat. MAB6194). Rabbit anti-human αvβ8 mAb EM13309 (IgG, Absolute Antibodies, cat. Ab00892) was also used together with control rabbit IgGs (Abcam, cat. ab37415). Mouse anti-mouse/human αvβ8 mAb ADWA-11 (IgG1) was generously provided by Dr. Dean Sheppard (University of California, San Francisco, CA, USA).

Secondary antibodies included Alexa Fluor 488-labeled goat anti-mouse antibodies (Invitrogen, cat. A-11001), Alexa Fluor 488-labeled goat anti-rabbit antibodies (Invitrogen, cat. A-11034), HRP-labelled goat anti-mouse Fc polyclonal antibody (Chemicon, Millipore, cat. #AP127P), and HRP-labelled goat anti-human IgG Fc (Southern Biotech, cat. 2014-05). Anti-mouse PD-L1 mAb 10F.9G2 was from Bio-Cell (cat. BE0101). Horseradish peroxidase (HRP)-labelled streptavidin (STV-HRP) (cat. S5512), normal goat serum (NGS, cat. 6767), and bovine serum albumin were from Sigma. Endoproteinase Lys-C (cat. 11047825001) was from Roche Diagnostics.

Recombinant human integrins α5β1 (cat. 3230-A5B), αvβ8 (cat. 4135-AV), αvβ6 (cat. 3817-AV), αvβ3 (cat. 3050), αvβ1 (cat. 6579-AVB), and αIIbβ3 (cat. 7148-A2-25) were purchased from Bio-Techne. Biotinylated recombinant human αvβ8 and αvβ6 were from ACROBiosystems (cat. IT8-H82W5 and cat. IT6-H82E4). Human TGFβ1 was from InvivoGen (cat. rcyc-htgfb1).

The composition of the buffers used in this study is described in **Table S1**.

### Peptides

Peptides were purchased from InnoPep (CA, USA) or from Proteogenix (France) or were prepared in-house by chemical synthesis 21, 22 (see **Table S2**). Peptides were dissolved in sterile water, aliquoted, and maintained at -20 °C until use. Peptide concentrations were determined using the BCA assay (Thermo Fisher Scientific, A55864), the Ellman’s assay (Thermo Fisher Scientific, 22582), or both, as appropriate. Peptide **5a** concentration, supplied by InnoPep, was also determined by elemental analysis and quantification of its net content.

### Production of the peptibodies (4ΔmFc and 4ΔhFc)

Cloning, expression, and partial purification of peptibodies were performed by Proteogenix (France). cDNAs coding murine and human peptibodies (called **4Δ**mFc and 4**Δ**hFc, respectively) were prepared by chemical synthesis (*see* **Figure S1** and **Figure S2**). These constructs were designed to encode a) a human heavy chain IgG1 signal secretion peptide, b) the CgA-derived peptide FETLRGDLRILSILRHQNLLKEL (called peptide **4Δ**), c) a four-glycine spacer, and d) a murine or human IgG_1_-Fc fragment. Both cDNAs were subcloned into the pTXs1 expression plasmid (ProteoGenix) by seamless cloning procedures. The resulting plasmids were used to transfect XtenCHO mammalian cells (ProteoGenix, cat. 10130938) using the Mammalian Protein Expression Kit (Proteogenix, cat. PX-XTE-001). Transfected cells were grown in Xten CHO Expression Medium (Proteogenix, cat. PX-XTE-002) in Erlenmeyer baffled flasks for 10-14 days (see **Figure S3 and Figure S4**). After centrifugation, the supernatant was harvested, sterile-filtered (0.22 µm), diluted 1:1 in *PBS-Sigma*, and loaded onto a HiTrap Protein A HP column (Cytiva, cat. 17040201). Bound proteins were eluted from the column with 20 mM citric acid (pH ~ 2.7) and immediately neutralized with 1 M Tris-HCl, pH 9.0. The eluates were then dialyzed against *PBS-Sigma,* aliquoted, frozen, and shipped to our laboratory for further purification. After thawing, samples were concentrated by ultrafiltration (Vivaspin 20 Ultrafilter 10kDa MWCO, Cytiva, cat. 28932360) and further purified by gel filtration on HiLoad Superdex 200 peptide grade column (Cytiva, cat. 28989335). Fractions showing a hydrodynamic volume of ~60 kDa were pooled, filtered (0.22 µm), and aliquoted. Yields were 60-80 mg/L culture. The purity of the final products was >95%, as determined by SDS-PAGE and densitometric analysis.

### Mass spectrometry analyses

Molecular weight and identity of the peptibodies were assessed using two complementary strategies: a) a top/middle-down approach to determine the intact mass, structural integrity, and N-glycosylation pattern, and b) a bottom-up approach to confirm full sequence coverage.

#### Top/middle-down approach

The intact murine peptibody was analyzed by MALDI-TOF/TOF mass spectrometry (RapifleX™ TissueTyper™, Bruker Daltonics) as described previously 23. Data were acquired in linear positive mode (range: 20-200 kDa), with the M5-Thin layer laser setting. Bovine serum albumin was used for external calibration. Raw spectral data were analyzed using flexAnalysis (Version 4.2; Build 14; Bruker Daltonics) applying baseline correction and smoothing, followed by manual peak assignment.

For the human peptibody, intact, reduced, and reduced/deglycosylated forms were analyzed at Evotec (Toulouse, France) as follows: the sample was reduced with dithiothreitol (200 mM, 30 min at 37 °C) and deglycosylated with N-glycosidase F (PNGase F) (enzyme-to-substrate ratio 1:5, 3 h at 37 °C). The resulting products (intact, reduced, and deglycosylated peptibody) were then analyzed by LC-MS using a C4 reverse-phase column connected to a UPLC (Acquity, Waters) and analyzed on a Q-TOF instrument (Waters) (scan range 200–4000 m/z) with collision-induced dissociation.

#### Bottom-up approach

Murine peptibody (10 µg) was reduced with dithiothreitol (10 mM final concentration, in 50 mM ammonium bicarbonate, 50 µL final volume, 1 h, 56 °C), and subsequently alkylated with iodoacetamide (27 mM, final concentration, 30 min at room temperature). The resulting product was then digested with endoproteinase Lys-C (enzyme-to-substrate ratio 1:50, w/w, 16 h, 37°C). The digested sample was then analyzed by nLC-MS/MS using a home-made C18 reverse-phase nanoLC column (75 μm i.d. x 15 cm, packed with ReproSil-Pur 120 C18-AQ resin, 1.9 μm) (Dr. Maisch HPLC) connected to a nUPLC Vanquish Neo (Thermo Fisher Scientific). Peptide elution was carried out using the following chromatographic conditions: *buffer A*, 0.1% v/v formic acid in water; *buffer B*, acetonitrile with 0.1% v/v formic acid; linear-gradient 0%–35% B over 45 min, flow rate: 300 nL/min. Eluted peptides were ionized via nano-electrospray (nano-ESI, Proxeon Biosystems) and analyzed using a Q-Exactive high-resolution mass spectrometer (Thermo Fisher Scientific) in data-dependent acquisition mode. MS1 spectra were acquired over the 300–2000 m/z range at a resolution of 70,000 with the 10 most abundant ions selected for fragmentation by higher-energy collisional dissociation at 27% normalized collision energy. Data analysis was performed using Mascot software (version 2.6, Matrix Science) against the expected peptibody sequence, allowing carbamidomethylation of cysteine as a fixed modification, methionine oxidation, and N-terminal acetylation as variable modifications, 5 ppm tolerance for MS1 and 0.02 tolerance for MS2, semi-LysC as proteolytic enzyme; 2 missed cleavages allowed.

### Peptibody-integrin binding assays

The capability of peptibodies to bind various integrins (αvβ1, αvβ3, αvβ6, αvβ8, α5β1, αII_b_β3 and αvβ8) was evaluated using three distinct assays, including a) a direct assay based on peptibody binding to microtiter plates coated with integrins, followed by detection with HRP-labeled anti-Fc Ab (*Assay 1*), b) a competitive assay based on competitive binding of peptibody with an isoDGR-HRP conjugate (a probe for the RGD-binding site of integrins) to integrin-coated plates (*Assay 2*), and c) a sandwich assay based on microtiter plates coated with **4Δ**mFc, biotinylated αvβ8 or αvβ6, and streptavidin–HRP conjugate (*Assay 3*). A schematic representation of each assay is shown in **Figure 2**.

#### Direct integrin binding assay (Assay 1)

Microtiter plates (96-well, cat. 675061, Greiner Bio-One) were coated overnight at 4 °C with or without human integrins (αvβ8, αvβ6, αvβ3, αvβ1, or α5β1) in DPBS (Euroclone, cat. EC-B4053, 2 µg/mL, 50 µL/well). After removal of the coating solution, plates were filled with bovine serum albumin (BSA, 3% w/v in DPBS, 150 µL/well) and incubated for 2 h at r.t. Following this, the microtiter plates were then washed with 20 mM Tris-HCl, pH 7.4, containing 1 mM MgCl_2_, 1 mM MnCl_2_, 150 mM NaCl, and 0.05% Tween-20 *(Buffer-1)* and filled with different peptibody concentrations (50 µL/well) in *Buffer-1* supplemented with 1% BSA and 1% v/v normal goat serum (NGS) (*Binding Buffer-1*). After incubation (1.5 h), each plate was washed with *Buffer-1* and subsequently filled with an HRP-labelled goat anti-mouse Fc polyclonal antibody (Chemicon, Millipore, cat. #AP127P, 50 µL/well, 1:2000 in *Binding Buffer-1*) and incubated for 1 h at room temperature. After six rapid washes, the plates were then filled with *Buffer-1* and incubated for 5 min; this process was repeated once. The solution was then removed, and the plates were filled with *Buffer-1* and incubated for an additional 5 min*.* After emptying the plate, the bound peroxidase was detected by adding the o-phenylenediamine chromogenic substrate (OPD, Sigma, cat. P-6912, prepared as recommended by the manufacturer). Ten minutes later, the absorbance at 490 nm was measured. The binding of **4Δ**mFc to integrins was calculated by subtracting the non-specific binding to wells lacking integrins. Binding curves were then fitted in GraphPad Prism using the “*one-site specific binding*” model to estimate dissociation constant (*K_D_*). The interaction of the human peptibody was studied essentially as described above, except that a secondary goat anti-human Fc polyclonal antibody (Southern Biotech, cat. 2014-05, 1:2000, 50 µL/well) was used to detect the binding.

#### Competitive integrin binding assays (Assay 2)

*Assay 2* was conducted as previously described 21, 24. In brief, increasing concentrations of peptibody were mixed with the isoDGR-HRP conjugate at a fixed concentration, and then transferred to plates coated with integrins. After incubation (2 h), each plate was washed. Bound peroxidase activity was detected using the OPD chromogenic substrate. The inhibitory constants (*Ki*) were determined using the “*One site-Fit Ki*” model in GraphPad Prism software as previously described 21.

#### Direct integrin binding assay (Assay 3)

Polyvinyl chloride microtiter plates (96-well, Carlo Erba, cat. FA5280100) were coated overnight at 4 °C with or without **4Δ**mFc in DPBS (5 µg/mL, 50 µL/well). After washing with 20 mM Tris-HCl, pH 7.4, containing 1 mM MgCl_2_, 150 mM NaCl, and 0.05% Tween-20 (*Buffer-2*), the microtiter plates were filled with *Buffer-2* supplemented with BSA (2% w/v) (200 µL/well) and incubated at 37 °C for 1.5 h. The microtiter plates were washed and filled with increasing concentrations of biotinylated αvβ8 or αvβ6 integrins (50 µL/well) diluted in *Buffer-2* supplemented with BSA (0.5% w/v) (*Binding Buffer-2*). After incubation (1 h), the microtiter plates were washed and filled with STV-HRP conjugate (50 µL/well, 1:2000 in *Binding Buffer-2*, 1 h). After washing, bound peroxidase activity was detected by adding 3,3′,5,5′-tetramethylbenzidine-phenylenediamine (chromogenic substrate, Sigma, cat. T3405). Ten min later, the absorbance at 450 nm was measured. Binding curves were fitted in GraphPad Prism using the “*one-site specific binding*" model.

### Surface plasmon resonance analysis

Dissociation constants (K_D_) of **4Δ**hFc interactions with αvβ6 or αvβ8 were measured by Surface-Plasmon Resonance (SPR) using a Biacore 8K+ instrument as described in the Supporting Information.

### Peptibody-cell binding assays

The binding of **4Δ**mFc to human T3M-4 cells was analyzed by FACS of detached cells after incubation with the peptibody, as well as by fluorescence analysis of cultured adherent-living cells incubated with peptibody-IRDye conjugates.

#### FACS analysis

The binding of **4Δ**mFc to human T3M-4 cells was analyzed by incubating cells, detached with trypsin-EDTA, with different amounts of peptibody (range: 0-100 nM) in 25 mM HEPES buffer, pH 7.4, containing 1 mM MnCl_2_, 1 mM MgCl_2_, 150 mM NaCl, 0.2% sodium azide, and 2% w/v BSA (*Binding Buffer-3*) (1 h on ice or at 37 °C). After washing, the cells were incubated for 1 h on ice with a goat anti-mouse Alexa Fluor 488-labeled secondary antibody (5 µg/mL in *Buffer-3*). After further washings, the cells were fixed with paraformaldehyde (2% in PBS) and the fluorescence bound to cells was measured using a flow cytometer (Accuri C6, Beckman Coulter). Flow cytometry data were analyzed using FlowJo software (BD Biosciences).

#### Binding of Peptibody-IRDye conjugates to cultured living cells

**4Δ**mFc and murine recombinant IgG1 Fc fragments (mFc, a control isotype, Abcam, cat. ab316088) were conjugated, via their amine groups, to IRDye800 CW (a near infrared fluorescent dye, herein designated as IRDye) using the Protein Labeling Kits (LI-COR, P/N: 928-38044) according to the manufacturer’s instructions (using a dye/protein ratio, 3:1). The unreacted dye was removed by gel filtration chromatography using a Zeba™ desalting spin column (Pierce) and DPBS without Ca/Mg as eluent (EuroClone, cat. EC-ECB4004L). The resulting conjugates were called **4Δ**mFc/IRDye and mFc/IRDye, respectively. A negative control conjugate, Cys/IRDye, with a cysteine residue in place of protein, was also prepared 22. The binding of **4Δ**mFc/IRDye to adherent wild-type and ITGB6/B8-KO T3M-4 cells was analyzed as follows: cells were grown in microtiter plates (96-well, Greiner Bio-one, cat. 655090, 2–3×10^4^ cells/well, 200 µL/well, seeded 24-48 h before the experiment). After washing with 0.9% NaCl solution, the cells were incubated with 25 mM Hepes buffer, pH 7.4, containing 150 mM NaCl, 1 mM MgCl_2_, 1 mM MnCl_2_, and 1% BSA *(Binding Buffer-4)* for 5 min. Peptibody-IRDye conjugates (range: 0–6 nM in *Binding Buffer-4*) were then added to the cells and incubated for 1 h (37 °C, 5% CO_2_). After three additional washings with *Binding Buffer-4* (200 μl/well, 5 min each), the cells were incubated with PBS supplemented with 3% sucrose and 2% paraformaldehyde (15 min at r.t). The binding of conjugates to cells was quantified by scanning the plate (filled with DPBS, 100 μl/well) with a Sapphire Biomolecular Imager scanner (Azure Biosystems, model Sapphire RGBNIR) using the following settings: *scan intensity*, 8; *scan focus*, 2.5 mm; *scan speed*, highest; *filter ex/em*, 784/832BP37; *Pixel size*, 75 μm. Bound fluorescence was quantified using Image Studio™ (Licor, version 5.5.4).

### Peptibody-tissue binding assays

The binding of **4Δ**mFc to human PDAC, lung adenocarcinoma, and colon carcinoma tissue sections was investigated by immunohistochemical and immunofluorescence techniques. Human surgical samples were collected at the Pathology Unit of the San Raffaele Hospital (Milan, Italy) for routine diagnostic or monitoring purposes and used under written informed consent. The study was conducted in accordance with the Declaration of Helsinki and approved by the Ethical Committee of San Raffaele Hospital. The surgical specimens were embedded in an optimal cutting temperature (OCT) compound and stored in the institutional biobank at the Biological Resource Center (CRB-OSR). Tissue samples were sliced (5-7 µm), air-dried (1-24 h), and incubated with *Binding Buffer-4* (10 min), followed by the same buffer containing 5 µg/mL of **4Δ**mFc or MOPC-31C mAb (a murine IgG1 isotype control). After 1 h of incubation, the sections were washed with the same buffer (2 x 5 min) and incubated with an HRP-labelled goat anti-mouse antibody (SignalStain® Boost IHC Detection Reagent, Cell Signaling, cat. 8125) (1:350, in *Binding Buffer-4*, 30 min). After washing with the same buffer*,* the binding of peptibody was detected using the DAB substrate (Biocare Medical, cat. BDB2004L) prepared according to the manufacturer’s instructions. The sections were then fixed in 10% neutral buffered formalin (Thermo Scientific, cat. 5701) for 5 min, and then rapidly washed with 1X Tris-Buffered Saline (Bio-Rad cat. 70-6435) and incubated in the same buffer for 5 min. Finally, the sections were counterstained with hematoxylin (Bio-Optica, cat. M06004), dehydrated, and mounted according to standard procedures.

For αvβ6 co-localization studies, fresh-frozen OCT-embedded lung adenocarcinoma sections were cut, air-dried, and blocked as described above. The sections were then incubated with a mixture of **4Δ**mFc (5 µg/mL) and the rabbit anti-αvβ6 mAb E4M9P (Cell Signaling, cat. #95153S, 1:200) in *Binding Buffer-4* (1 h, room temperature). After washing with *Binding Buffer-4* (2 × 5 min, each), the sections were incubated with a mixture of a donkey anti-mouse Alexa Fluor 555 (Invitrogen, cat. A21127) and a donkey anti-rabbit Alexa Fluor 488 (Life Technologies cat. A21206) secondary antibodies (1:350 and 1:500, respectively, in *Binding Buffer-4*, 1 h). After extensive rinsing, the sections were mounted with Vectashield antifade mounting medium containing DAPI (Vector Laboratories, cat. H-1200-10) and analyzed using confocal microscopy using either a TCS SP5 (Leica Microsystems) or a FluoView FV3000RS (Olympus), as indicated in the figure captions.

### TGFβ bioassay

The effect of **4Δ**mFc on TGFβ activation was assessed as previously described 22, using human bladder cancer 5637 cells and murine PDAC 5M701/GFP/CEA cells, i.e., two cell lines that can produce active TGFβ 23. The conditioned medium of these cells was analyzed using HEK-Blue™ TGFβ cells (InvivoGen, cat. hkb-tgfbv2), a reporter cell model designed to detect bioactive TGFβ.

### Conjugation of proteins to a pH-Sensitive Dye (pHsDye)

The **4Δ**mFc and mFc were coupled to NHS-ester pHAb (Promega, cat. G98414), an activated form of dye that reacts with primary amines and fluoresces in acidic environments. Conjugation was performed in PBS (pH 7.2) for 16 h at 4 °C using a protein/dye molar ratio of 1:7 (~2.94 nmol of protein mixed with ~20.4 nmol of dye, in 285 µL final volume). The reaction mixture was then purified using an NAP-5 column (Cytiva; eluent, PBS) to remove the unreacted dye. Similarly, a goat anti-human Fc polyclonal antibody (Southern Biotech, cat. 2014-01) and a goat anti-mouse IgG polyclonal antibody (Sigma, cat. M8642) were labeled with the same dye using a protein/dye molar ratio of 1:5 or 1:15, respectively, using 10 mM sodium borate, pH 9.0, as coupling buffer. The resulting conjugates were called **4Δ**mFc-pHsDye, mFc-pHsDye, anti-mouse IgGs-pHsDye, and anti-human Fc-pHsDye. The protein concentration of each conjugate and the degree of labeling (DOL) were determined by measuring the absorbance at 280 and 532 nm, respectively, according to the manufacturer’s instructions. The results showed that **4Δ**mFc-pHsDye and mFc-pHsDye had a DOL of ~1, while secondary pAbs had a DOL of ~1-3.5.

### Cell internalization assay

The internalization of protein-pHsDye conjugates by BxPC-3, LN-229, and T3M-4 cells was analyzed as follows: cells were grown in 96-well microtiter plates (Greiner, cat: 655090, 3-4×10^4^ cells/well, 100 µL/well, seeded 24 h before the experiment) in cell culture medium. On the day of the assay, the cell culture medium was replaced with DMEM FluoroBrite (Gibco, cat. A1896701), 1% glutamine, 1% penicillin/streptomycin, and 10% FBS (*internalization buffer*) without or with protein-pHsDye conjugates (0.10-200 nM). After 18-20 h of incubation at 37 °C and 5% CO_2_, the medium was removed, the cells were washed with DPBS without Ca/Mg, fixed with DPBS containing 3% sucrose, 2% paraformaldehyde, for 15 min at r.t., emptied, and filled with DPBS (100 µL/well). The conjugate internalization was then quantified by scanning the wells with a Sapphire Biomolecular Imager system (Sapphire RGBNIR, Azure Biosystems; excitation filter, 520 nm; emission filter, 565 nm (BP24 nm); intensity, *7 or 8*; speed, *highest*; resolution, *50 µm*; focus, *2.5* mm).

Peptibody internalization was also assessed using complexes of **4Δ**mFc or **4Δ**hFc with pHsDye-labelled secondary pAbs (peptibody/secondary pAb ratio, 1:3). Controls with mFc or hFc in place of peptibodies were also prepared. The complexes (10-30 nM peptibody) were added to BxPC-3 cells for 20 h, and the internalized fluorescence was evaluated as described above.

The internalization of **4Δ**mFc complexed with secondary pAb-pHsDye conjugate was also evaluated using live-cell confocal fluorescence microscopy as follows: cells were incubated with the complex for 20 h (37 °C, 5% CO_2_), washed, and then counterstained for 15 min (37 °C, 5% CO_2_) with a mixture of LysoTracker™ Deep Red (25 nM final concentration; Thermo Fisher, cat L12492) and Hoechst 33258 (1 µg/mL, final concentration). Images were acquired in confocal mode using an ImageXpress system (Molecular Devices, San Jose, CA, US; 40× objective; excitation/emission filters: 531/40 and 593/40 nm for *pHsDye*; 631/28 and 692/40 for *LysoTracker™ Deep Red*; 377/54 and 447/60 nm for Hoechst 33258). Similar experiments were also conducted with the **4Δ**mFc-pHsDye direct conjugate (100 nM). In this case, cell-associated fluorescence was acquired using a confocal microscope (TCS SP5).

### *In vitro* cytotoxicity assay

Double-positive αvβ6/αvβ8 cell lines (BxPC-3, T3M-4), αvβ8 single-positive cells (LN-229), and β6/β8 integrin double-knockdown cells (ITGB6/B8KO T3M-4) were seeded in a 96-well plate (Cambrex, cat. LT27-102) (2500-7500 cells/well, 100 µL/well). After 4 h at 37 °C and 5% CO_2_, cells were treated with serial dilutions of a pre-assembled complex consisting of **4Δ**hFc and a secondary antibody anti-human Fc drug conjugate (ADC) (Moradec, cat. AH-101AF-50 (non-cleavable MMAF), cat. AH-102AF-50 (cleavable MMAF), cat. AH-102DD-50 (cleavable MAAE), cat. AH-102PN-50 (cleavable duocarmycin DM), cat. AH-102PN-20 (cleavable PNU159682), cat. AH-103D1-50 (non-cleavable DM1), cat. AH-106PB-50 (cleavable PBD), cat. AH-107DX-50 (cleavable DX8951), prepared in complete cell medium (180 nM **4Δ**hFc and 270 nM ADC, 10 min at r.t.). The complexes were diluted 1:5 and added to the cells (150 µL/well final volume). After 72-144 h, cell viability was determined using the CellTiter-Glo™ 2.0 kit (Promega, cat. G9242) and an EnSpire Alpha plate reader instrument (Perkin Elmer) using a dedicated built-in protocol for luminescence detection. Data were analyzed using GraphPad Prism software with a nonlinear 4-parameter curve-fitting model.

### *In vivo* studies in animal models

The procedures of *in vivo* studies in mice were approved by the Animal Care and Use Committee of S. Raffaele Hospital Animal Facility and by the Italian Ministry of Health (IACUC numbers 1565). The studies were performed at the San Raffaele Hospital (an authorized institution) in accordance with institutional guidelines and national and international laws and regulations. Particular care was taken to ensure animal welfare, with a focus on minimizing the number of animals and their suffering.

Female BALB/c mice (18-20 g, Charles River Laboratories) were subcutaneously injected in the left flank with either 3×10^5^ TS/A cells or 1.5×10^6^ cells WEHI-164 fibrosarcoma. Tumor growth was monitored by measuring tumor size with calipers. The tumor volume was estimated using the formula (r1×r2×r3×4/3π), where r1 and r2 are the longitudinal and lateral radii, and r3 is the tumor thickness protruding from the normal skin surface. Tumor volumes are shown as mean ± SE. Mice were sacrificed before tumors reached 1-1.5 cm in diameter, or a volume of ≥800-1000 mm^3,^ or if extensive tumor ulceration or distress signs were observed. Mice were treated, i.p., with peptibody alone (35-50 µg/mouse in 0.9% NaCl, containing 100 μg/mL of HSA) or in combination with anti-PD-L1 mAb (10 mg/kg, i.p. in 0.9% NaCl) as indicated in the corresponding figures.

The infiltration of immune cells in tumors and the quantification of TGFβ in tumor extracts were performed by FACS analysis and ELISA techniques as described in the Supporting Information.

## Results

### Production of peptibodies

**Figure 1:**
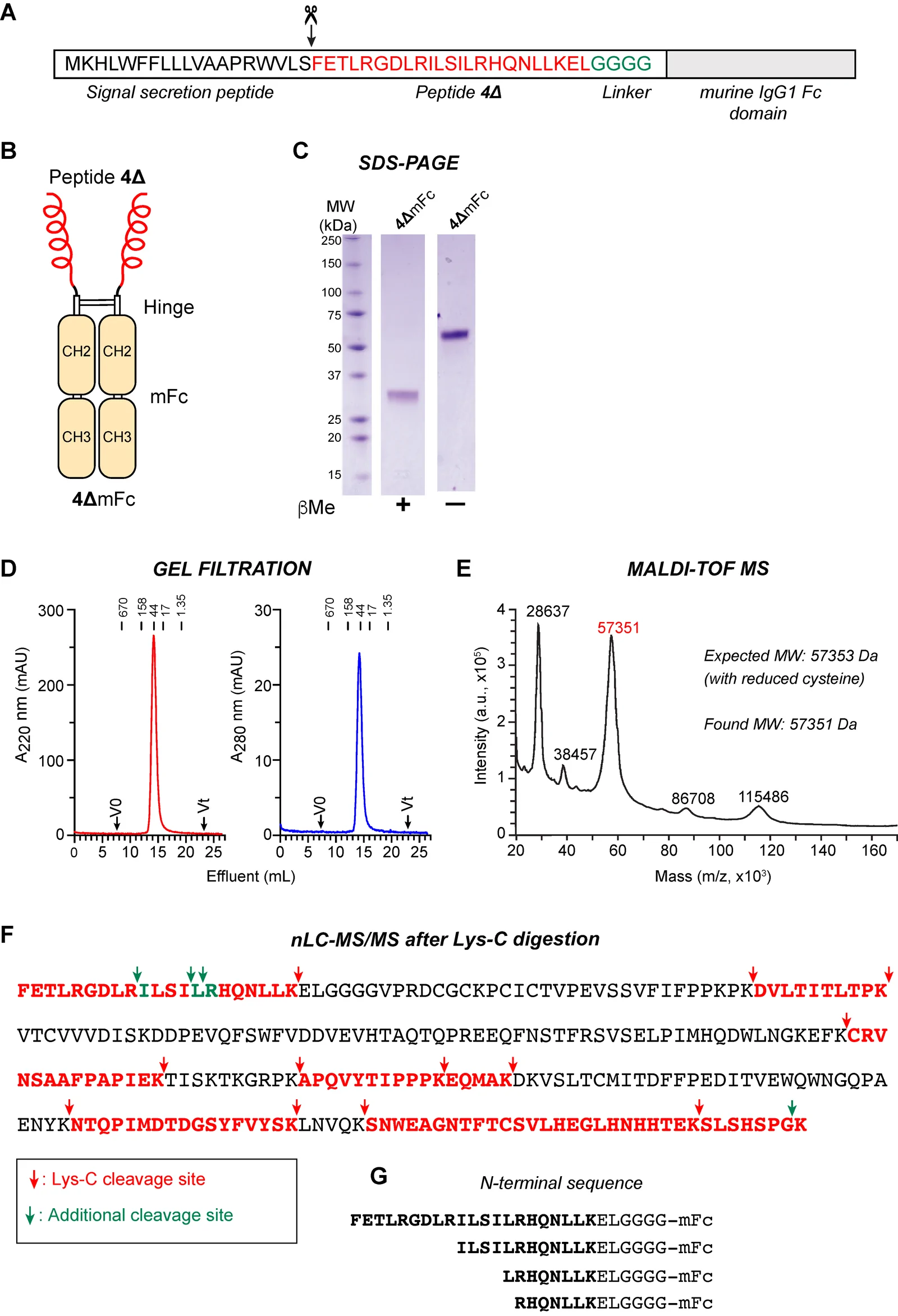
** Biochemical characterization of the peptibody 4ΔmFc. A**) Schematic representation of the **4Δ**mFc components, which include: a signal secretion sequence derived from a human heavy chain IgG1 (black sequence), the peptide **4Δ** (*bold-red sequence*), a four-glycine spacer linker (*green*) fused to a murine IgG1-Fc domain consisting of hinge plus CH2 and CH3 domains (*grey box*, sequence not shown). Amino acids are reported using the single-letter code. The *arrow* indicates the expected cleavage site of the signal secretion peptide. **B**) Schematic representation of the **4Δ**mFc structure. **C**) SDS-PAGE analysis of purified peptibody **4Δ**mFc under reducing (βMe +) and non-reducing (βMe –) conditions. About 1 µg of peptibody per lane was loaded. *MW*, molecular weight markers. **D**) Analytical gel-filtration chromatography of **4Δ**mFc on a Superdex-200 (10/300 GL) column. *Vo*, void volume; *Vt,* total volume; and the elution volumes of molecular-weight standards (670, 158, 44, 17, and 1.35 kDa) are indicated.** E**) MALDI-TOF mass spectrum of **4Δ**mFc. The molecular masses of the major components and the expected molecular weight of **4Δ**mFc calculated from the cDNA sequence (not glycosylated and with reduced cysteines) are indicated. **F**) Amino acid sequence of **4Δ**mFc as determined by nano liquid chromatography coupled with tandem mass spectrometry (nLC-MS/MS) after digestion with Lys-C, a protease that cleaves peptide bonds at the carboxyl side of lysine residues (red arrows). The peptides identified by nLC-MS/MS (bold red) cover 43% of the expected sequence. Green arrows and green residues mark additional cleavage sites not dependent on Lys-C digestion, likely reflecting pre-existing fragments and peptibody heterogeneity. **G**) N-terminal sequences of **4Δ**mFc peptibody components.

We produced, by recombinant DNA technology, a protein consisting of FETLRGDLRILSILRHQNLLKEL (a CgA-derived peptide called **4Δ**) fused to the Fc domain of a murine IgG1 (mFc) (see **Figure 1A-B,** and **Figure S1**). The resulting peptide-Fc fusion product (peptibody) was called **4Δ**mFc. This peptibody was transiently expressed in XtenCHO mammalian cells as a secreted protein and purified from the cell culture medium by protein-A affinity chromatography followed by gel-filtration chromatography (yield: about 60-80 mg/l of culture). SDS-PAGE analysis of the product, under reducing and non-reducing conditions, showed bands of ~30 and ~60 kDa, respectively, likely corresponding to the one- and two-chain forms of the protein (**Figure 1C**). The purity of the product was >95%, as determined by the densitometric analysis of the gels (**Figure 1C**). Analytical gel filtration chromatography of the product shows the presence of a single peak with an apparent molecular weight of about 60 kDa (**Figure 1D**). Mass spectrometry (MS) analysis of the intact **4Δ**mFc (by MALDI-TOF) showed a molecular weight of 57353 Da (expected ~ 60 kDa, **Figure 1E**). Moreover, nanoliquid chromatography-tandem mass spectrometry (nLC-MS/MS) analysis of the product after digestion with Lys-C, a proteolytic enzyme that cleaves peptide bonds after lysine (Lys, K), revealed fragments consistent with the expected peptibody sequence (**Figure 1F-G**). Notably, this biochemical technique revealed, in addition to molecules with the intact N-terminal sequence, minor components corresponding to molecules lacking part of the N-terminal sequence (FETLRGDLR, FETLRGDLRILSI, or FETLRGDLRILSIL), which likely correspond to degradation products formed during the production procedure.

The peptide **4Δ** was also fused to the Fc domain of a human IgG1 (hFc) to generate the corresponding human peptibody (**4Δ**hFc). This product was produced and purified using a method similar to that used for the murine peptibody, with a yield of 60 mg/liter of culture (*see* **Figure S2 and S4**). The molecular identity and integrity of the purified peptibody were checked by liquid chromatography coupled to electrospray ionization mass spectrometry (LC-ESI-MS) before and after reduction with dithiothreitol, and after reduction and deglycosylation with N-glycosidase F (PNGase F, which removes glycans linked to asparagine in consensus sequence Asn-X-Ser/Thr) (**Figure S5**). The results showed that the major MS component protein corresponds to the expected peptibody (~80%) bearing G0F glycoforms and lacking C-terminal lysine, a typical feature of recombinant IgG1 Fc domains (**Figure S5A**). Minor components (<20%) lacking part of the targeting peptide domain were also detected, consistent with the murine construct (**Figure S5A**). These findings were additionally confirmed for both reduced and reduced/deglycosylated samples (**Figure S5B**). Overall, these findings demonstrate that the production of murine and human peptibodies, with the peptide **4Δ** fused to an IgG1 Fc domain, is feasible.

### 4ΔmFc binds recombinant human αvβ6 and αvβ8

**Figure 2:**
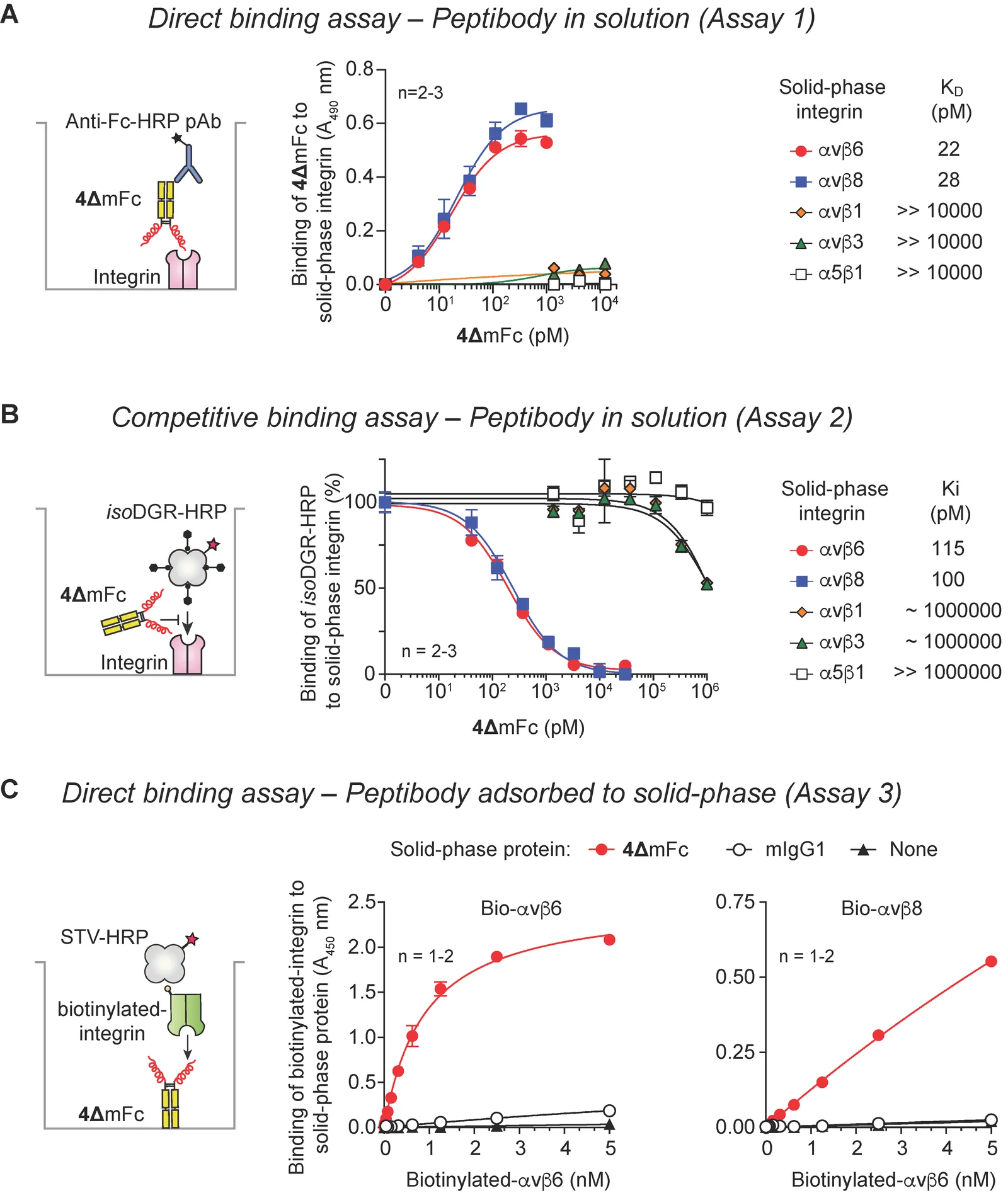
** Binding of peptibody 4ΔmFc to recombinant human αvβ1, αvβ3, αvβ6, αvβ8, and α5β1.** Schematic representation of the assay formats (*left panels*) and representative dose-response curves (*middle-right panels*) are shown. **A**) Direct binding of **4Δ**mFc to microtiter plates coated with the indicated integrins, as detected using an HRP-labeled goat anti-mFc polyclonal antibody (*Anti-Fc-HRP pAb*). Non-specific binding, i.e., the binding of **4Δ**mFc to bovine serum albumin-coated wells, was subtracted to determine specific binding. **B**) Competitive binding of **4ΔmFc** and *iso*DGR-HRP conjugate to microtiter plates coated with the indicated integrins. **C**) Direct binding of biotinylated-αvβ6 or -αvβ8 to microtiter plates coated with **4Δ**mFc, murine IgG1 (mIgG1, negative control) or diluent (*None*), as measured using a streptavidin–HRP conjugate (STV-HRP). Dots represent mean ± SE of n = 1-3 wells (for some data points, error bars are smaller than the size of the symbol). Dissociation (*K_D_)* and inhibition (*Ki*) constants were calculated using “*one-site specific binding”* and “*one site-Fit Ki*” equations, respectively, in GraphPad Prism software.

The capability of **4Δ**mFc to recognize purified human integrins was investigated using direct and competitive binding assays. Direct integrin-binding assays, based on the use of microtiter plates coated with various human integrins, showed that **4Δ**mFc binds purified αvβ6 and αvβ8 with high affinity (22 and 28 pM, respectively). At the same time, very low binding, or no binding at all, was observed to αvβ1, αvβ3, and α5β1 (**Figure 2A** and** Table 1,** *Assay 1*). Similar results were obtained using competitive binding assays based on the use of isoDGR-HRP conjugate as a probe for the RGD binding site of all these integrins (**Figure 2B** and** Table 1,** *Assay 2*). To further evaluate binding specificity, another assay was performed based on the use of **4Δ**mFc or a control IgG1 adsorbed to the solid phase and biotinylated integrins (bio-αvβ6 and bio-αvβ8) in the liquid phase. As expected, both integrins could bind the peptibody, but not the control IgG1 (**Figure 2C,** *Assay 3*), confirming that the peptibody-integrin interactions occurred through the peptide moiety and not the Fc domain. Overall, these results suggest that **4Δ**mFc can bind the RGD-binding site of αvβ6 and αvβ8 with high affinity and selectivity.

The high affinity of 4ΔhFc for αvβ6 and αvβ8 was also confirmed using an independent biophysical method based on Surface Plasmon Resonance (SPR). While the avidity set-up revealed very strong apparent binding (apparent K_D_ <1 nM) (**Figure S6A**), the affinity set-up, designed to minimize multivalent effects, showed low-nanomolar binding to αvβ6 and αvβ8 (K_D_=1.5 and 2.6 nM, respectively) (**Figure S6B**). No binding was observed for control hFc. These results validate the integrin-binding specificity of the 4Δ moiety and confirm high-affinity recognition of both αvβ6 and αvβ8.

### 4ΔmFc binds to cultured αvβ6/αvβ8-positive cancer cells

**Figure 3:**
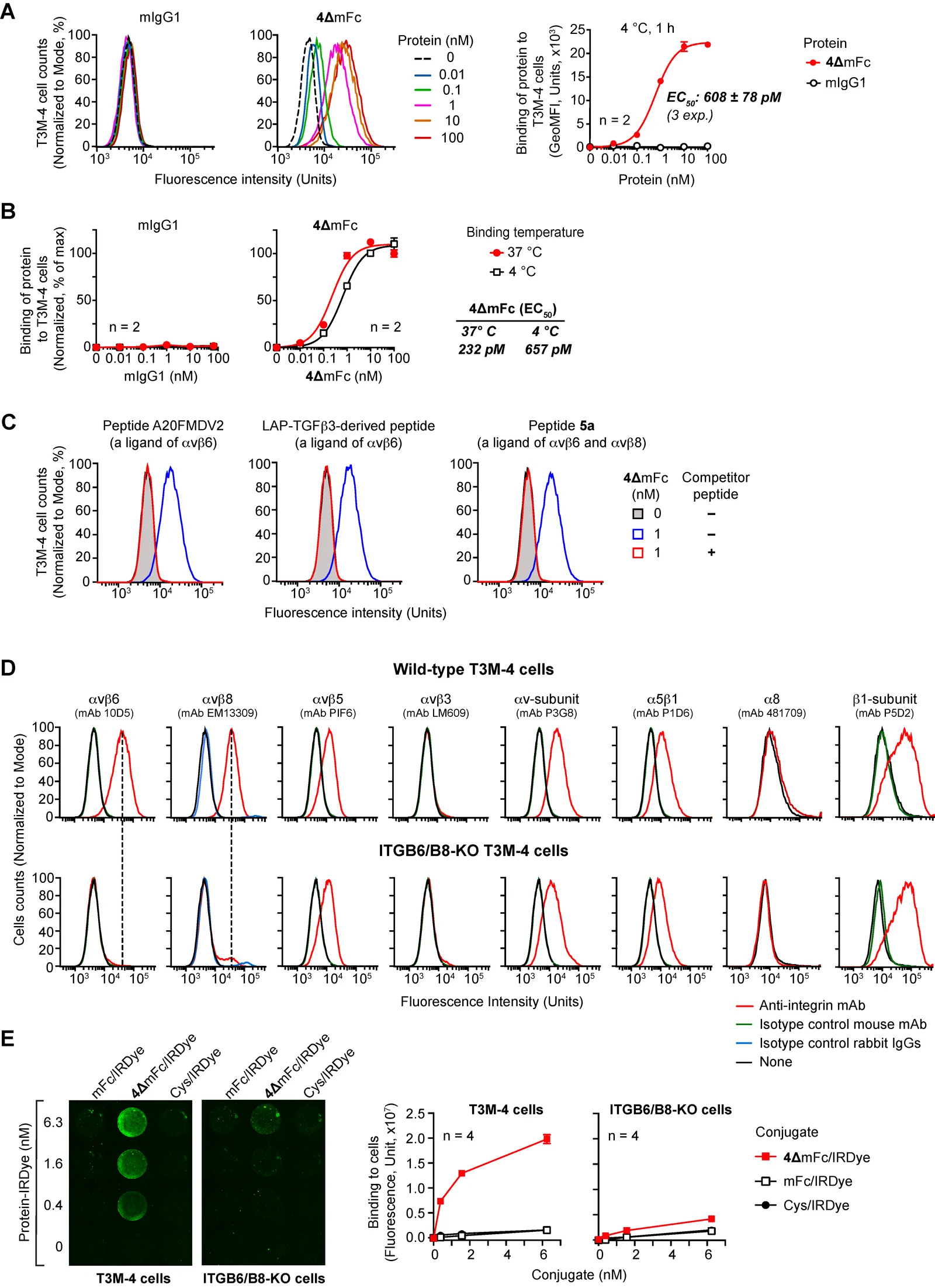
** Binding of peptibody 4ΔmFc to human T3M-4 pancreatic ductal adenocarcinoma cells. A**) Binding of **4Δ**mFc and of the murine antibody MOPC-31C (isotype control, mIgG1) to αvβ6/αvβ8-positive T3M-4 cells at 4 °C, as detected by FACS analysis using a goat anti-mouse Alexa Fluor 488-labeled secondary antibody (*left and middle panels*). Dose-response binding curve (*right panel*). Dots, mean ± SE of 2 technical replicates. The effective concentration 50 (EC_50_) of **4Δ**mFc, determined with three independent experiments, is also shown (mean ± SE). **B**) Effect of the temperature on the binding of **4Δ**mFc to T3M-4 cells as determined by FACS analysis. Cells were incubated with various amounts of **4Δ**mFc at 4 °C or 37 °C for 1 h. After cell washing, bound **4Δ**mFc was detected as described above. **C**) Competition of **4Δ**mFc binding to T3M-4 cells with *i*) peptide A20FMDV (a known ligand of αvβ6), *ii*) ac-HGRGDLGRLKK-amide peptide, derived from LAP-TGFβ3 (a ligand of αvβ6), or *iii*) peptide **5a** (a bispecific ligand of αvβ6 and αvβ8). One nanomolar **4Δ**mFc was mixed with the indicated peptides (100 nM) and added to T3M-4 cells. After 1 h of incubation on ice, the cells were washed, and bound **4Δ**mFc was detected by FACS as described above. **D**) Expression of αvβ6, αvβ8, αvβ5, αvβ3, αv-subunit, and α5β1 on T3M-4 (*upper panels*) and on β6/β8 double-knockout T3M-4 cells (ITGB6/B8-KO cells, *lower panels)* as determined by FACS using the indicated primary antibodies, followed by Alexa Fluor 488-goat anti-rabbit or goat anti-mouse IgG polyclonal secondary antibodies. Dotted lines represent the αvβ6 and αvβ8 expression levels of wild-type cells. **E**) Binding of **4Δ**mFc labelled with IRDye800 (**4Δ**mFc/IRDye), to wild-type T3M-4 and ITGB6/B8-KO T3M-4 cells. Cells were incubated with the indicated amounts of **4Δ**mFc/IRDye or with negative controls (a murine Fc or cysteine labeled with the same dye, called mFc/IRDye and Cys/IRDye, respectively). After 1 h of incubation at 37 °C/5% CO₂, cells were washed and fixed with formaldehyde; cell-bound fluorescence was then quantified using an Azure Biosystems scanner (see *Methods*). Representative images of microtiter wells seeded with the indicated cells and treated with the indicated amounts of conjugates (*left panels*); conjugate binding curves (mean ± SE of 4 wells) (*right panels*). In *Panels A and B*, dots represent the mean ± SE of the indicated technical duplicates (for some data points, error bars are smaller than the size of the symbol).

The capability of **4Δ**mFc to recognize αvβ6/αvβ8-positive cancer cells was then investigated. To this aim, human T3M-4 PDAC cells (αvβ6^+^/αvβ8^+^) were incubated with various concentrations of **4Δ**mFc, at 4 °C and 37 °C; then, the binding of peptibody to cells was analyzed by FACS using a fluorescein-labeled anti-mouse IgG antibody. The results showed that **4Δ**mFc, but not a control IgG1, could bind T3M-4 cells with an EC_50_ of about 600 pM (**Figure 3A**); a lower EC_50_ value (about 200 pM) was obtained when the assay was performed at 37°C (**Figure 3B**). Notably, the binding was inhibited by an excess of known ligands of αvβ6 and αvβ8, such as peptide **5a** 21, peptide A20FMDV2 25, or a TGFβ3-derived peptide (_ac-_HGRGDLGRLKK-_NH2**,**_ 26), but not by peptide **2a**, a control CgA-derived peptide with RGE in place of RGD (**Figure 3C**). These results suggest that **4Δ**mFc can recognize human αvβ6/αvβ8-positive cancer cells and that its RGD sequence is crucial for binding.

To verify that the binding to cells was indeed mediated by αvβ6 and αvβ8 integrins, we performed additional peptibody-cell binding assays using wild-type T3M-4 PDAC cells (αvβ6^+^ and αvβ8^+^) and αvβ6/αvβ8 double-knockout cells (ITGB6/B8-KO) (αvβ6^–^/αvβ8^–^, **Figure 3D**). The peptibody **4Δ**mFc, but not the mFc fragment or Cys (negative controls), all labeled with the fluorescent dye IRDye800, could bind wild-type T3M-4 cells. In contrast, little or no binding was observed with ITGB6/B8-KO cells (**Figure 3E**). These findings confirm the hypothesis that peptibody-cell binding occurs through the interaction of the **4Δ** moiety with αvβ6 and αvβ8. Notably, no binding of peptibody occurred to ITGB6/B8-KO cells, despite these cells express α5β1, αvβ5, α8β1, αv- and β1-subunits (**Figure 3D**). These findings agree with the binding data obtained with purified integrins, confirming that the peptibody is selective for αvβ6 and αvβ8.

### 4ΔmFc binds to αvβ6-positive human tumor tissue sections

**Figure 4:**
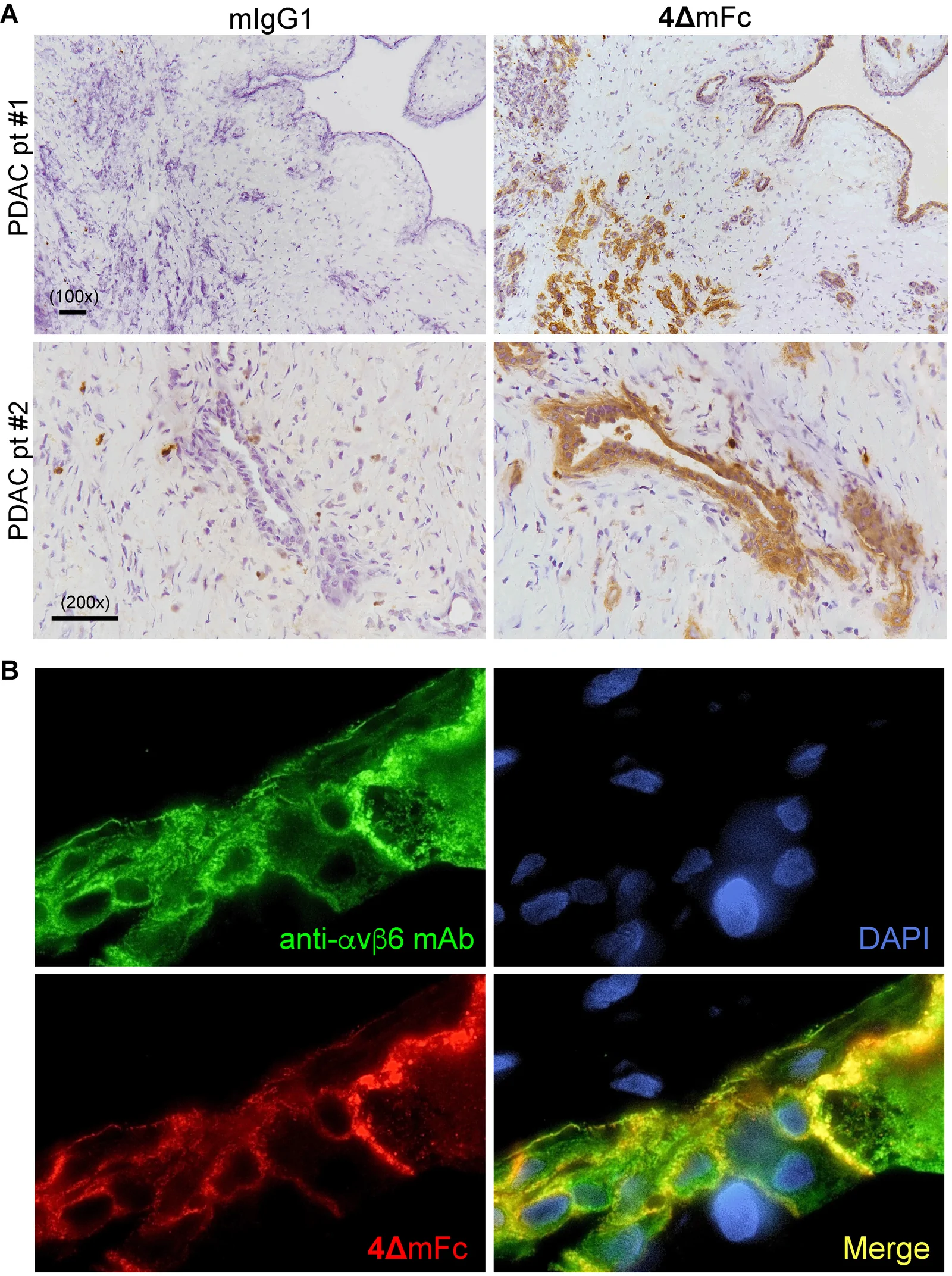
** Binding of peptibody 4ΔmFc to fresh-frozen human pancreatic ductal adenocarcinoma (PDAC) and lung adenocarcinoma (ADCA) tissue sections. A**) Binding of **4Δ**mFc (*right*) or mAb MOPC-31C (isotype control, mIgG1) (*left*) to PDAC tissue sections from two patients (pt), as detected by immunohistochemistry using an HRP-labelled goat anti-mouse antibody and DAB chromogen (*brown color*). Tissue sections were counterstained with hematoxylin. Scale bars and magnification are shown. **B**) Binding of a rabbit anti-αvβ6 mAb (*green*) and **4Δ**mFc (*red*) to a lung ADCA tissue section as detected by immunofluorescence analysis using a donkey anti-rabbit Alexa Fluor 488 and donkey anti-mouse Alexa Fluor 555 antibodies. Sections were counterstained with 4',6-diamidino-2-phenylindole (DAPI, *blue*). Microphotographs were acquired using a confocal microscope (TCS SP5, Leica Microsystems) at 400× magnification.

To assess whether **4Δ**mFc can bind integrins expressed in human tumors, we analyzed the binding of this peptibody to human PDAC, lung adenocarcinoma, and colon carcinoma tissue sections, i.e., tumors known to overexpress αvβ6 8, 10, 11, 27, by immunohistochemical and immunofluorescence techniques. The results showed that **4Δ**mFc, but not a control IgG1 isotype (mAb MOPC-31C), could efficiently stain cancer cells in these tumor histotypes (**Figure 4A-B,** and **Figure S7A**). Of note, the binding of **4Δ**mFc to lung and colon cancer cells co-localized with that of anti-αvβ6 antibodies (**Figure 4B** and **Figure S7A**). Importantly, in colon cancer tissue sections, an excess of free peptide **4Δ** efficiently abolished the staining of **4Δ**mFc, indicating that the **4Δ** moiety of peptibody is critical for the binding and that peptide **4Δ** and peptibody compete for the same integrin-binding site (**Figure S7B**). These results, taken together, suggest that **4Δ**mFc can also recognize human integrins in tumors.

### 4ΔmFc inhibits the production of active TGFβ by cancer cells

**Figure 5:**
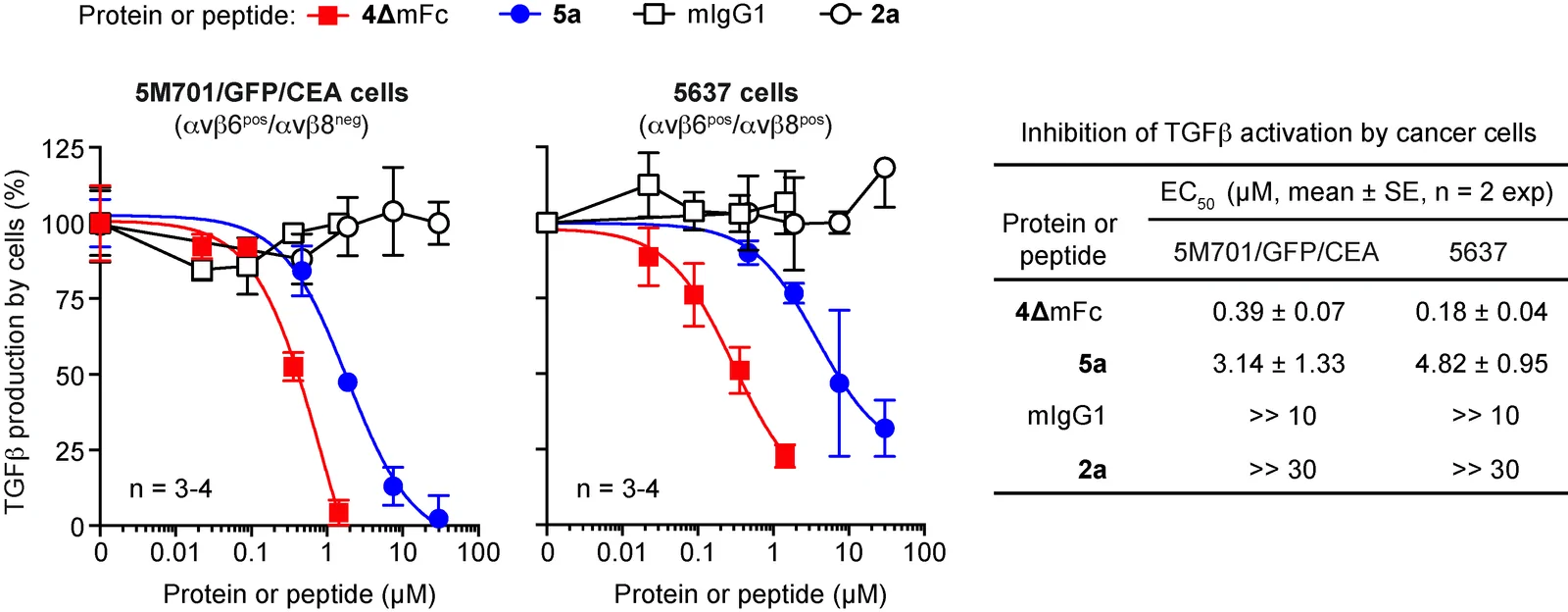
** 4ΔmFc and peptide 5a, but not mIgG1 and peptide 2a (negative controls) inhibit TGFβ-activation mediated by 5M701/GFP/CEA and 5637 cancer cells.** Cells were plated in 96-well microtiter plates, left to adhere (2 h), and exposed to the indicated proteins or peptides for 24-48 h. The concentration of active TGFβ in the supernatants was assessed using a bioassay based on HEK-Blue™ TGFβ cells, as detailed in the *Methods*. A representative experiment out of two performed is shown (mean ± SE, n = 3-4 wells). The EC_50_ of each compound, calculated from the results of both experiments, is shown.

To assess whether **4Δ**mFc can inhibit TGFβ activation mediated by αvβ6 or αvβ8-positive cancer cells, we then investigated the effect of this peptibody on TGFβ production by murine 5M701/GFP/CEA PDAC cells (αvβ6^+^/αvβ8^–^) and 5637 bladder cancer cells (αvβ6^+^/αvβ8^–^), i.e., two cell lines that can produce active TGFβ 23. In parallel, a control isotype murine IgG1 mAb and peptide **2a** were used as negative controls, while peptide **5a** was used as a positive control. The amount of active TGFβ in cell supernatants was quantified after 24-48 h incubation using a bioassay based on HEK-Blue™ TGFβ cells. As expected, **4Δ**mFc and **5a**, but not the IgG1 control isotype and peptide **2a**, could inhibit the activation of TGFβ by these cells in a dose-dependent manner (**Figure 5**). Remarkably, **4Δ**mFc was about 8-14-fold more potent than the free peptide **5a**, suggesting that peptide dimerization via Fc increased its binding avidity. These results indicate that **4Δ**mFc can interact with αvβ6/αvβ8-positive cancer cells and, consequently, block their capability to maturate TGFβ through integrin-mediated mechanisms.

### 4ΔmFc is efficiently internalized by PDAC and GBM cells via αvβ6/αvβ8 integrin recognition and traffics into the lysosomal compartment

The internalization properties of **4Δ**mFc were next investigated. To this end, we coupled **4Δ**mFc or mFc (negative control) to a pH-sensitive fluorescent dye (pHsDye), a compound that fluoresces in acidic compartments (as in endosomes and lysosomes). UV/VIS spectrophotometric analysis of the conjugates (called **4Δ**mFc-pHsDye and mFc-pHsDye) showed a degree of labeling (DOL) of approximately one molecule of dye per protein molecule (DOL: ~1).

**Figure 6:**
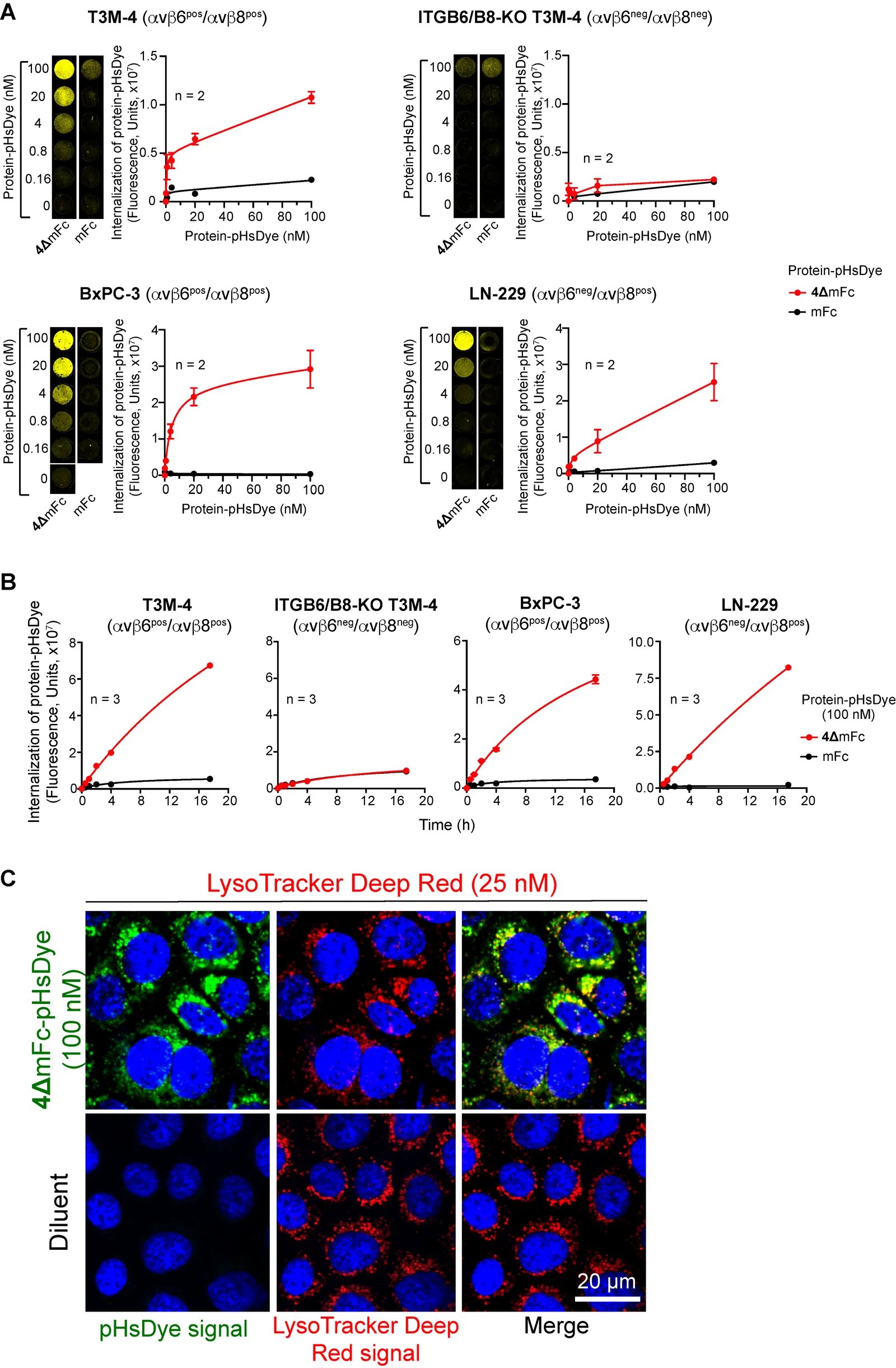
** 4ΔmFc, but not control mFc, is efficiently internalized into αvβ6- and/or αvβ8-expressing cell lines and traffics into the lysosomal/endosomal compartments. A**) Internalization of **4Δ**mFc-pHsDye and mFc-pHsDye in wild-type T3M-4, ITGB6/B8-KO T3M-4, BxPC-3, and LN-229 cells. Various concentrations of **4Δ**mFc-pHsDye or control mFc/pHsDye were added to 96-well microtiter plates seeded the previous day with the indicated cells. After 18-20 h of incubation at 37 °C and 5% CO₂, cells were washed, and cell-associated fluorescence was acquired with a Sapphire scanner. Representative images of microtiter wells are shown; the yellow pseudocolor indicates intracellular fluorescence. Dots, mean ± SEM of 2 wells (for some data points, error bars are smaller than the size of the symbol). **B**) Internalization kinetics of **4Δ**mFc-pHsDye and mFc-pHsDye. **4Δ**mFc-pHsDye or mFc/pHsDye (100 nM) was incubated with the indicated cells for 0, 0.5, 1, 4, and 17.5 h. Internalized fluorescence was then measured as described in *panel A*. Dots, mean ± SEM of 3 replicate wells (error bars are smaller than the size of the symbol). **C**) **4Δ**mFc-pHsDye is efficiently internalized into the lysosomal/endosomal compartments. **4Δ**mFc-pHsDye (100 nM) or diluent was added to BxPC-3 cells and incubated overnight at 37 °C, 5% CO₂. Cells were then washed and stained with LysoTracker Deep Red (25 nM) to label lysosomes/endosomes and with Hoechst 33258 to stain the nuclei. After a final wash, cell-associated fluorescence was acquired using a confocal microscope (TCS SP5, Leica Microsystems) equipped with a 63× objective. Single-channel and merged images (digitally magnified 3x) are shown for LysoTracker Deep Red (*red*) and protein-pHsDye conjugates (*green*). Hoechst 33258 fluorescence (*blue*) is displayed in all images. Note that the pHsDye signal is displayed in green to distinguish it from LysoTracker deep red, whereas in *panel A* it is pseudocolored in yellow to enhance contrast on a dark background.

The internalization of these conjugates in BxPC-3 and T3M-4 cells (αvβ6^+^/αvβ8^+^), ITGB6/B8-KO T3M-4 cells (αvβ6^-^/αvβ8^-^), and LN-229 cells (αvβ6^-^/αvβ8^+^) was then investigated (**Figure S8**). **4Δ**mFc-pHsDye was efficiently internalized, in a dose- and time-dependent manner, by BxPC-3, LN-229, and T3M-4 cells, but not by ITGB6/B8-KO cells (**Figure 6A-B**). In contrast, mFc-pHsDye exhibited minimal uptake by all cell lines, consistent with its lack of targeting specificity. Internalization of **4Δ**mFc-pHsDye was clearly detected after 0.5 h, and the fluorescence signal increased over time without reaching saturation even after ~18 h **(Figure 6B)**. This non-saturating uptake profile is consistent with that reported for other therapeutic mAbs, including trastuzumab and cetuximab, labeled with the same pHsDye 28. To further validate the internalization capability of peptibody, we performed a pulse-chase assay in T3M4 cells, using **4Δ**mFc/IRDye and control mFc/IRDye. After removal of unbound conjugate at different time points, the fluorescence signal of **4Δ**mFc/IRDye bound to cells rapidly declined during the first 8 h and then reached a plateau, with approximately 20–30% of the initial signal remaining associated to the cells for up to 72 h (**Figure S9**). Collectively, these results demonstrate that **4Δ**mFc is efficiently bound, internalized and retained by cells expressing αvβ6/αvβ8. Notably, the fluorescence signal overlapped with that of LysoTracker™ Deep Red, a fluorescent marker specific for lysosomes, pointing to receptor-mediated internalization of the complexes and trafficking to the lysosomal compartment (**Figure 6C**).

### 4ΔhFc mediates delivery and internalization of imaging or therapeutic cargos in αvβ6/αvβ8-positive cells

The capability of murine and human peptibodies to deliver and internalize imaging or therapeutic cargos was then investigated. To this aim, **4Δ**mFc, **4Δ**hFc, mFc, or hFc were complexed with anti-murine IgG or anti-hFc antibodies labelled with pHsDye. **4Δ**mFc and **4Δ**hFc, but not mFc or hFc, efficiently internalized these cargos in BxPC-3, LN229, and T3M-4, but not in ITGB6/B8-KO T3M-4 cells (**Figure S10A-B**). These findings demonstrate that both murine and human peptibodies can efficiently mediate the internalization of large protein assemblies, such as antibody conjugates, and deliver them to lysosomal compartments, as confirmed by their colocalization with LysoTracker™ Deep Red (**Figure S10C**).

**Figure 7:**
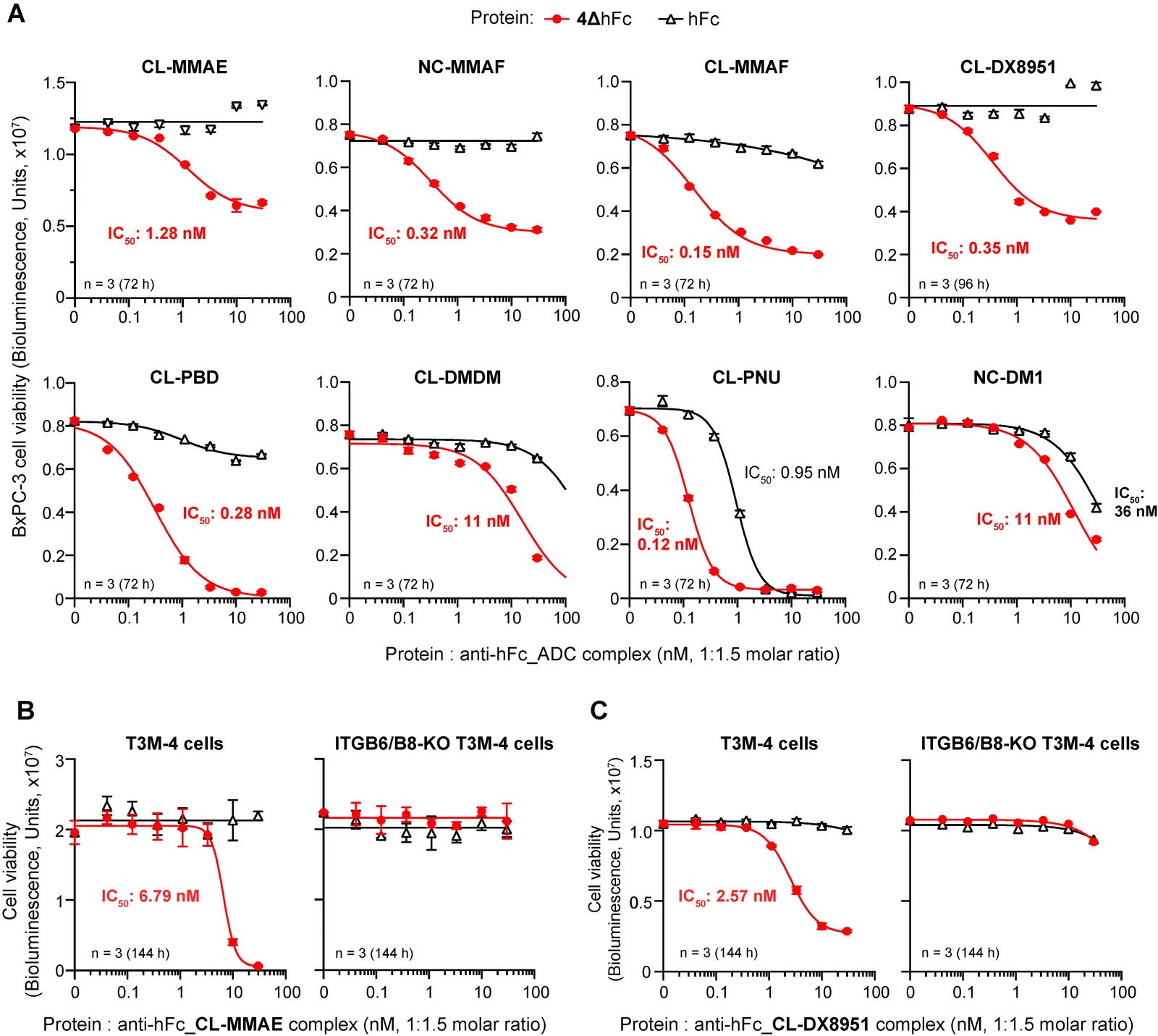
** 4ΔhFc enables targeted delivery of cytotoxic payloads to BxPC-3 and T3M-4, but not to ITGB6/B8-KO cells. 4Δ**hFc or control hFc were pre-mixed with an anti-human Fc secondary polyclonal antibody conjugated with either cleavable (CL) or non-cleavable (NC) linkers to the following payloads: *MMAE* (monomethyl auristatin E), *MMAF* (monomethyl auristatin F), *DX8951* (exatecan mesylate), *PBD* (pyrrolobenzodiazepine), DMDM (duocarmycin DM), *PNU* (PNU-159268) or *DM1* (emtansine). Mixtures were prepared at a 1.5-fold molar excess of secondary ADC over the peptibody and added to the indicated cells seeded 4 h before the assay (2500-7500 cells/well). After incubation for the indicated times, cell viability was measured by the CellTiter-Glo™ 2.0 assay. **A-C**) Dose-response curves for BxPC-3 cells (7500 cells/well) incubated with the indicated peptibody-ADC complexes (**A**), for T3M-4 and ITGB6/B8-KO cells (2500 cells/well) incubated with peptibody-ADC complexes containing cleavable MMAE (**B**) or cleavable DX8951 (**C**). Dots, mean ± SEM of *3* replicate wells (for some data points, error bars are smaller than the size of the symbol). The IC₅₀ value for **4Δ**hFc, or control hFc, complexed with the indicated secondary ADC is indicated in each panel.

Thus, we evaluated the ability of the **4Δ**hFc to deliver cytotoxic drugs into target cells. To this aim, **4Δ**hFc was pre-complexed with anti-human Fc secondary antibodies coupled to different cytotoxic drugs, including microtubule inhibitors (MMAE, MMAF, DM1), a DNA alkylator (PBD), a topoisomerase I inhibitor (DX8951), and a DNA minor groove binder (DMDM). All **4Δ**hFc-complexes induced marked, dose-dependent reduction of BxPC3 cell viability, while hFc-complexes showed negligible or markedly lower cytotoxicity (**Figure 7A**). Notably, complexes made with **4Δ**hFc and MMAE, MMAF, DM1, or DX8951 showed IC₅₀ values ranging from 0.12 to 1.3 nM. Furthermore, cytotoxic effects were observed with **4Δ**hFc-MMAE or **4Δ**hFc-DX8951 complexes on αvβ6^+^/αvβ8^+^ T3M-4 cells (IC₅₀: ~6.8 nM and ~2.6 nM, respectively), but not on ITGB6/B8-KO cells (**Figure 7B-C**), pointing to αvβ6/αvβ8 recognition, complex internalization, and intracellular release of the payload.

### 4ΔmFc/IRDye has a terminal plasma half-life of about 3 days

To assess the plasma half-life of **4Δ**mFc, this product was labeled with the IRDye800 fluorochrome. The conjugate, called **4Δ**mFc-IRDye800, but not control mFc labeled with IRDye800 (mFc-IRDye800), could bind αvβ6-coated microtiter plates (**Figure S11A**), suggesting that the IRDye800 moiety does not impair the **4Δ**mFc integrin recognition properties. Thus, the 4ΔmFc-IRDye800 conjugate was administered (i.v.) to healthy C57BL/6 mice, and plasma samples were collected at various time points. Analysis of plasma fluorescence showed that this conjugate has a biphasic elimination profile, with a rapid early decline (~4 h) followed by a log-linear terminal phase consistent with a terminal half-life of ~3 days (**Figure S11B** and **Table S3**). Although these data reflect the half-life of the **4Δ**mFc/IRDye conjugate and not of the peptibody alone, they strongly suggest that peptide fusion to the Fc IgG fragment is a valid strategy to prolong its plasma half-life.

### 4ΔmFc inhibits tumor growth in the WEHI-164 fibrosarcoma model

**Figure 8:**
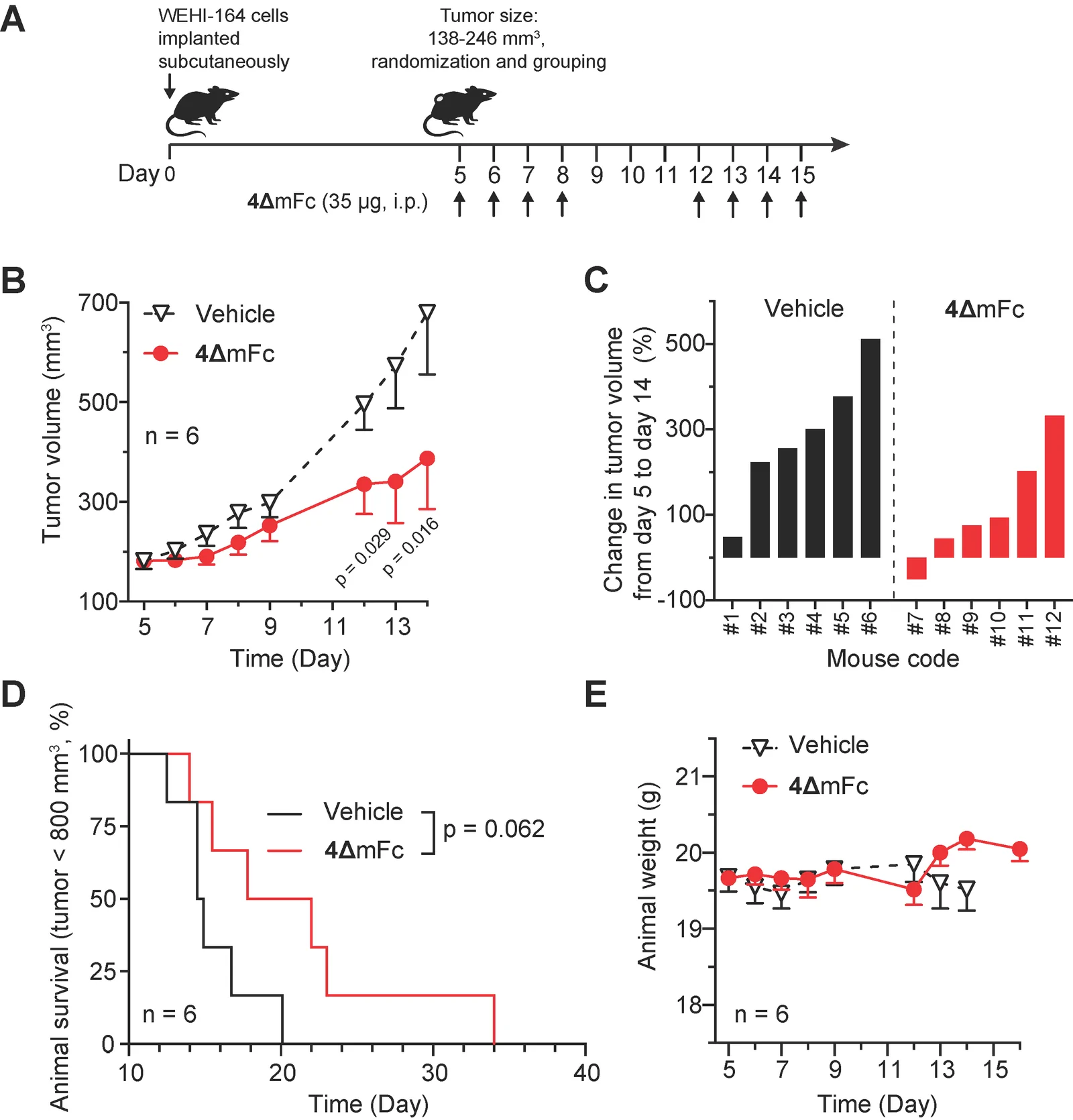
** 4ΔmFc inhibits the growth of subcutaneous lesions of WEHI-164 fibrosarcomas in mice. A**) Experimental scheme. WEHI-164 fibrosarcoma cells were injected, subcutaneously, into 12 mice. Five days after implantation, the mice were randomized into 2 groups (n = 6 mice/group). Mice were injected, intraperitoneally, with diluent (*Vehicle*) or with 35 µg of **4Δ**mFc (4 consecutive treatments/week for 2 weeks, totaling 8 treatments). **B**) Tumor volume curves (mean ± SE, n = 6 mice/group). Differences between groups were assessed at day 13 and 14 by a two-way ANOVA with post-hoc Sídák's multiple comparisons test; *P* values are shown. **C)** Waterfall plots showing the tumor volume variation (percentage) for each individual mouse over the indicated time. **D**) Kaplan-Meier survival curves. Statistical differences between groups (p=0.062) were assessed using the log-rank (Mantel-Cox) test. Mice were euthanized when tumors reached ≥ 800 mm^3^ or when extensive tumor ulceration or other signs of distress were observed. **E**) Animal body weight of tumor-bearing mice following treatment with the indicated compounds (mean ± SE, n = 6 mice/group).

The anti-tumor activity of 4**Δ**mFc was then evaluated in a murine model of fibrosarcoma, based on WEHI-164 cells (αvβ6^–^ and αvβ8^+(low)^) implanted subcutaneously in syngeneic BALB/c mice. Tumors were allowed to grow to 140-250 mm^3^; then mice were injected with **4Δ**mFc (35 µg/mouse, i.p.) four times per week for a total of 8 administrations (**Figure 8A**). This product significantly delayed the tumor growth and extended the survival of some mice (**Figure 8B-D and Figure S13A**). The antitumor activity of **4Δ**mFc was not associated with signs of toxicity, such as changes in animal body weight (**Figure 8E**), animal behavior, or fur aspect.

### 4ΔmFc exerts synergistic anti-tumor effects with an anti-PD-L1 monoclonal antibody

**Figure 9:**
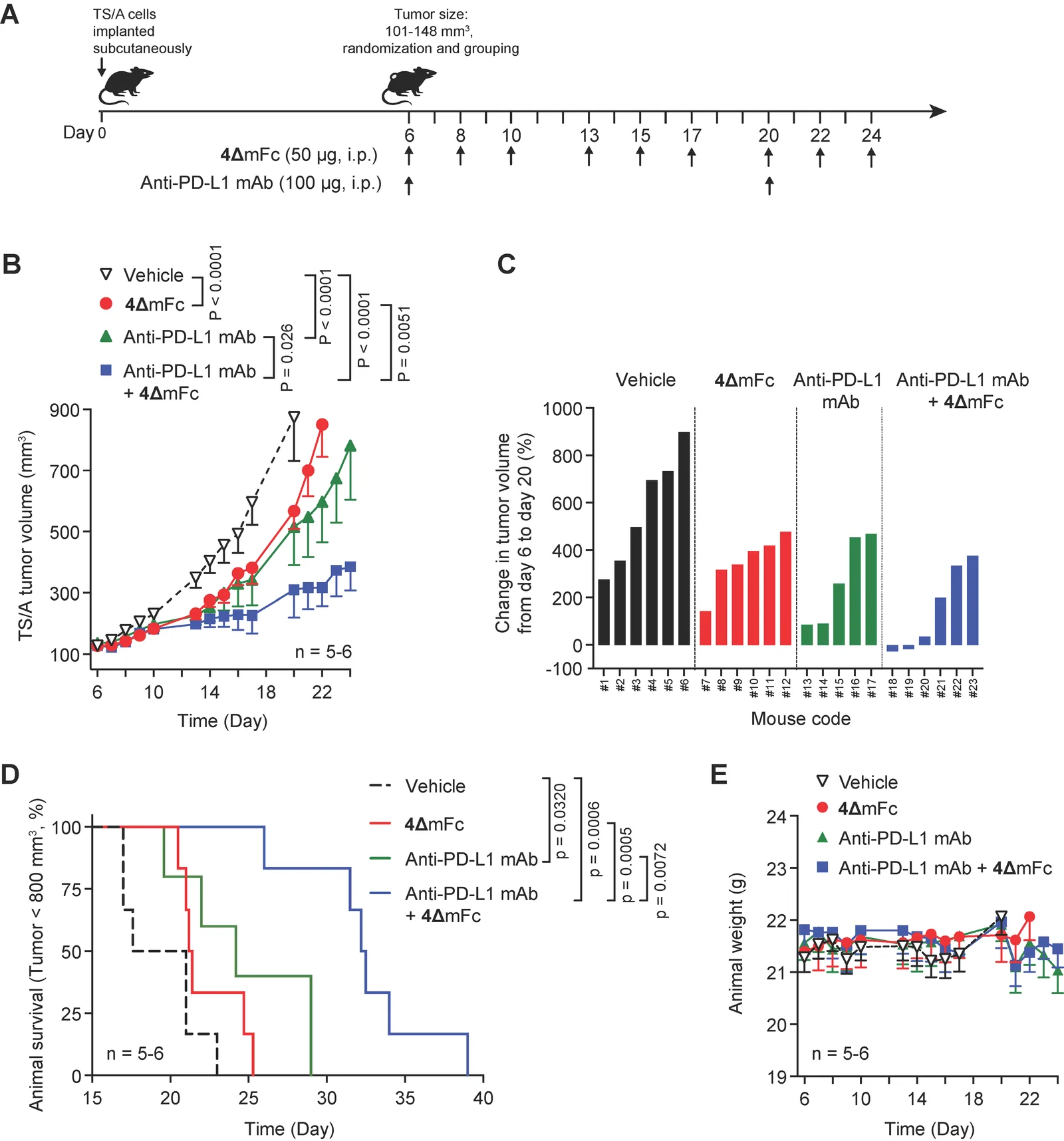
** Peptibody 4ΔmFc synergizes with anti-PD-L1 mAb to reduce TS/A tumor size and improve survival of tumor-bearing mice**. **A**) Experimental scheme. TS/A mammary adenocarcinoma cells were injected, subcutaneously, into 23 mice. Six days after implantation, the mice were randomized into four groups (n = 5-6 mice/group). Mice were injected intraperitoneally (i.p.) with diluent (*Vehicle*) or with 50 µg of **4Δ**mFc (3 treatments/week, administered every other day, for a total of nine treatments) or with 100 µg of an anti-PD-L1 mAb (on days 6 and 20) or a combination of **4Δ**mFc and anti-PD-L1 mAb (with anti-PD-L1 mAb administered 10 min before **4Δ**mFc). **B**) Growth curves of tumors (mean ± SE, n = 5-6 mice/group). Statistical differences between the groups were analyzed with GraphPad Prism software using a two-way ANOVA followed by Tukey's multiple comparisons test (from day 6 to day 20, when all the mice were alive); *P* values are shown. **C**) Waterfall plots showing the tumor volume variation (percentage) for each individual mouse over the indicated time. **D**) Kaplan-Meier survival curves. *P* values, calculated by long-rank Mantel-Cox test, are shown. Animals were euthanized when tumor volume reached 800-1000 mm^3^, or earlier if marked tumor ulceration or other signs of distress were observed. **E**) Body weight of mice bearing TS/A tumors after treatment with the indicated compounds (mean ± SE, n = 5-6 mice/group).

The anti-tumor activity of** 4Δ**mFc in combination with a modulator of the immune response, such as the anti-PD-L1 mAb 10F.9G2, was then investigated. Since TS/A mammary adenocarcinoma cells express the PD-L1 antigen (**Figure S12**), we decided to use these cells implanted subcutaneously in syngeneic mice for the study. The combined treatment (see **Figure 9A** for the treatment schedule) induced anti-tumor effects and increased mouse survival more efficiently than treatments with single compounds (**Figure 9B-C,** and **Figure S13B**). No evidence of toxicity, as judged from the loss of animal weight, was obtained in all groups (**Figure 9E**). These results suggest that** 4Δ**mFc exerts synergistic effects with anti-PD-L1 mAb, possibly by reducing immunosuppressive mechanisms in the tumor microenvironment.

### 4ΔmFc reduces TGFβ and enhances immune-cell infiltration in TS/A tumors

**Figure 10:**
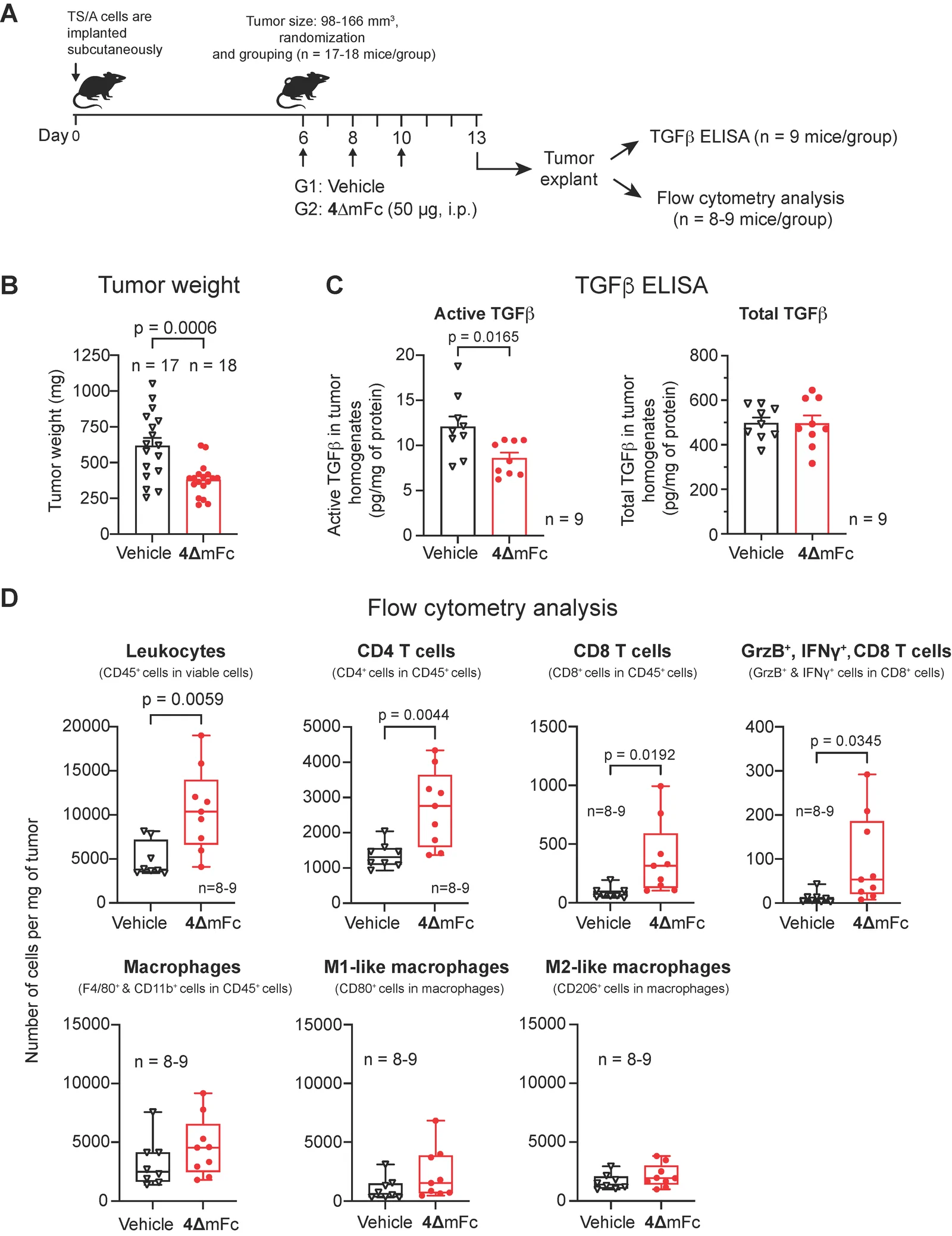
** 4ΔmFc reduces active TGFβ levels and enhances immune cell infiltration in the TS/A tumor microenvironment. A**) Experimental scheme. TS/A mammary adenocarcinoma cells were implanted subcutaneously in 35 mice. On day 6 after tumor implantation, tumor-bearing mice were randomized into two experimental groups and treated intraperitoneally with vehicle alone (Group G1: n = 17 mice) or with 50 µg of **4Δ**mFc (Group G2: n = 18 mice), at the times indicated by the arrows. Tumors were collected on day 13, weighed, and randomly assigned to either TGFβ quantification by ELISA (n = 9 tumors/group) or flow cytometry analysis (n = 8–9 tumors/group). **B**) Tumor weight. Bars, mean ± SE of n = 17-18 tumors/group. Dots represent individual tumors. *P* value, calculated by two-tailed unpaired t-test, is indicated in the plot. **C**) Active and total TGFβ levels in TS/A tumor homogenates as determined by ELISA. Bars, mean ± SE of n = 9 tumors/group. Dots represent individual tumors. *P* value was calculated by two-tailed unpaired t-test and is indicated in the plot. **D**) Flow cytometry quantification of tumor-infiltrating immune cells, expressed as cells per mg of tumor. Dots represent individual tumors; box plots indicate the median and interquartile range (n = 8-9 tumors/group). *P* values were calculated by two-tailed unpaired t-test and are indicated in the plots.

We next investigated the effect of **4Δ**mFc on active TGFβ levels in TS/A tumors and on immune-cell infiltration. To this aim 35 tumor-bearing mice were randomized in two experimental groups and systemically treated with **4Δ**mFc (50 μg/mouse, n = 18 mice) or vehicle (n = 17 mice/group), 3 administrations, every other day, for 1 week (see **Figure 10A**). Three days after the last treatment, the mice were sacrificed and tumors were explanted and weighed (**Figure 10B**). TGFβ levels in tumor extracts were then analyzed by ELISA (n = 9 mice/group); tumor immune-cell infiltration (n = 8-9 mice/group) was analyzed by flow cytometry.

**4Δ**mFc significantly reduced active TGFβ levels (**Figure 10C**) and increased the number of CD45^+^ leukocytes, CD4^+^ T cells, CD8^+^ T cells, cytotoxic effector CD8^+^ T cells (GrzB^+^, IFNγ^+^), compared to control treated mice (**Figure 10D**). No significant differences were observed in macrophages and M1- and M2-like macrophages (**Figure 10D**).

These findings indicated that **4Δ**mFc can reduce active TGFβ levels in tumors and enhance the infiltration and activation of cytotoxic effector CD8⁺ T cells.

## Discussion

The main finding of this study is that **4Δ**-derived peptibodies can be exploited as novel multifunctional platforms to inhibit TGFβ activation in tumors, deliver cytotoxic payloads to cancer cells, and enhance the efficacy of PD-L1 checkpoint blockade.

The results of biochemical and biological studies show that both murine and human peptibodies (**4ΔmFc** and **4ΔhFc,** consisting of peptide **4Δ - FETLRGDLRILSILRHQNLLKEL -** fused to the Fc domain of human or murine IgG1) efficiently recognize the RGD-binding site of αvβ6 and αvβ8 integrins, either in purified form or when expressed on the surface of cancer cells, with nanomolar affinity. Murine **4Δ**mFc can also efficiently recognize cancer cells in tissue sections from human pancreatic ductal adenocarcinoma, lung adenocarcinoma, and colon carcinoma, while it exhibits little or no binding to adjacent “*normal*” tissues.

Notably, the binding affinity of these peptibodies for αvβ6 and αvβ8 (sub-nanomolar) is comparable to, or even higher than, that of peptide **5a** (CFETLRGDLRILSILRX1QNLX2KELQD, chemically stapled with a triazole bridge between the propargylglycine (X1) and azidolysine (X2) residues) previously described 25. This observation indicates that chemical stapling of the amphipathic α-helix, which is necessary for high-affinity binding of peptide **5a**, is not required when the sequence is fused to an Fc domain via a GGGG linker. Because the amphipathic α-helix adjacent to the RGD motif is crucial for integrin selectivity and affinity, these data suggest that peptide **4Δ** can adopt a stable α-helical conformation when linked to the Fc antibody fragment through the GGGG sequence, despite the absence of the triazole bridge. Thus, the **4Δ**–GGGG sequence, which is composed entirely of standard amino acids, represents a valuable alternative to peptide **5a** for the generation of peptide–protein conjugates when recombinant DNA technologies are required.

We previously demonstrated that **5a**-HSA, a peptide–albumin conjugate prepared by chemical synthesis, inhibits TGFβ activation mediated by αvβ6⁺/αvβ8⁺ cancer cells and by αvβ8⁺ regulatory T cells, and reduces TGFβ signaling within neoplastic tissues. *In vivo* experiments showed that this conjugate can promote CD8⁺ T cell-dependent anti-tumor responses and enhance the anti-tumor efficacy of S-NGR-TNF, a tumor vasculature–targeted form of TNF, ultimately leading to tumor eradication in some mice 23. However, this conjugate has several drawbacks that limit its clinical application, either as a drug alone or as a platform for delivering cytotoxic compounds to tumors. First, **5a**-HSA, which is produced by chemical conjugation using the heterobifunctional cross-linker sulfo-SMCC, is a heterogeneous mixture of albumin molecules bearing variable numbers of linkers and peptide moieties per protein 23. Second, the HSA used to prepare the conjugate may consist of different albumin isoforms 29, thereby representing an additional source of molecular heterogeneity. Third, as HSA is purified from human plasma donors, the isoform composition of different lots may also vary. The molecular heterogeneity of this conjugate may have important implications in process reproducibility, lot-to-lot consistency, analytical characterization, pharmacokinetics, and toxicology, thus representing a major limitation for clinical development. The human peptibody **4Δ**hFc, which can be produced by recombinant DNA technology as a homogeneous divalent product, can potentially overcome several of these limitations.

The results of the present study also show that **4Δ**hFc is efficiently internalized by αvβ6/αvβ8-positive tumor cells, but not on ITGB6/B8-knockout cells. Thus, this peptibody could be used, in principle, as a vector for delivering cytotoxic compounds to cancer cells. This view is supported by the results of cytotoxicity assays, showing that **4Δ**hFc, but not Fc, can markedly increase the cytotoxic effects of anti-Fc antibodies coupled to different cytotoxic drugs (such as MMAE, MMAF, DX8951, PBD, DMDM, PNU, or DM1) on αvβ6/αvβ8-positive tumor cells, but not on ITGB6/B8-knockout cells. These data point to a receptor-mediated mechanism and support the concept that **4Δ**hFc can be exploited as an efficient platform for the development of peptibody-drug conjugates (PBDCs). Although this indirect approach based on peptibody complexes with anti-antibody-drug conjugates can be used for screening purposes and for obtaining a proof-of-concept, this modular approach is not intended for clinical use. Therefore, direct **4Δ**hFc-drug conjugates need to be prepared to assess conjugate pharmacokinetics, stability, penetration, efficacy and safety.

Finally, our results show that **4Δ**mFc can delay tumor growth in the WEHI-164 fibrosarcoma model and improve mice survival in the TS/A mammary adenocarcinoma model when combined with an anti-PD-L1 mAb (an immune checkpoint inhibitor), without overt toxicity. Considering that activation of TGFβ in the tumor microenvironment represents an important immunosuppressive mechanism, it is possible that inhibition of this mechanism by **4Δ**mFc contributed to the therapeutic effects observed in these models. According to this view, **4Δ**mFc could indeed reduce the levels of active TGFβ in TS/A tumors. This effect was associated with increased infiltration of CD45⁺ leukocytes, CD4⁺ and CD8⁺ T cells, including cytotoxic CD8⁺ T cells. Owing to the complexity of these models, we do not know whether the observed therapeutic effects depend on tumor-, immune-, and/or stromal cell targeting: further studies in models based on cancer cells that express both αvβ6 and αvβ8, single integrins, or none of them, may help to clarify this point. Furthermore, further studies aimed at assessing the risk of excessive TGFβ inhibition in normal tissue should also be performed.

Immune checkpoint inhibitors have achieved remarkable outcomes in certain cancer types 30, 31. However, despite these notable achievements, not all patients exhibit a favorable response to this type of drug, underscoring the need for novel combination strategies to enhance their therapeutic effectiveness 32, 33. The capability of our peptibodies to inhibit TGFβ activation in the tumor microenvironment may represent new drugs to be used in combination with this class of compounds.

## Conclusions

Collectively, our findings identify **4Δ**hFc as a first-in-class, bi-selective αvβ6/αvβ8-targeting molecule that can be used to block TGFβ activation in the tumor microenvironment. This peptibody can also be used, in principle, for delivering cytotoxic compounds and theranostic agents to tumors in concomitance with reduction of αvβ6/αvβ8-dependent immunosuppressive mechanisms. The potential multi-functionality of **4Δ**hFc provides a strong rationale for using this peptibody not only to reduce immunosuppression and reprogram the tumor microenvironment in combination with immune checkpoint blockade or other immunotherapies, but also as a novel scaffold for the targeted delivery of therapeutic and diagnostic agents to tumors. Finally, the murine peptibody can be exploited as an immunohistochemical reagent to assess the expression of functionally active αvβ6/αvβ8 integrins in human tumors (active in terms of peptide recognition), for example before treatment with peptibody-drug conjugates and/or during therapy, thereby guiding decisions on whether to continue the treatment or switch to alternative therapeutic strategies.

## Supplementary Material

Supplementary methods, figures and tables.

## Figures and Tables

**Table 1:** Binding affinity of chromogranin A-derived peptides and peptibody for human αvβ6 and αvβ8 integrin.

Compound	Code	K_D_ (pM) (by direct integrin binding assay, Mean ± SE)							
		αvβ6	αvβ8	αvβ1	αvβ3	αvβ5	α5β1	αII_b_β3	α8β1
Murine peptibody	4ΔmFc	29 ± 5 *(3)*^a^	31 ± 3 *(3)*	>>10^4 b^ *(1)*	>>10^4^ *(1)*	*>>*10^4^ *(1)*	>>10^4^ *(1)*	*>>*10^4^ *(1)*	*>>*10^4^ *(1)*
Human peptibody	4ΔhFc	39 ± 2 *(2)*	79 ± 9 *(2)*	*NA* ^ c^	*NA*	*NA*	*NA*	>>10^4^ *(1)*	>>10^4^ *(1)*
Human Fc	hFc	>>10^4^ *(1)*	>>10^4^ *(1)*	*NA*	*NA*	*NA*	*NA*	>>10^4^ *(1)*	>>10^4^ *(1)*
		
		*Ki* (pM) (by competitive integrin binding assay, Mean ± SE)							
		αvβ6	αvβ8	αvβ1	αvβ3	αvβ5	α5β1	αII_b_β3	α8β1
Murine peptibody	4ΔmFc	477 ± 123 *(7)*	791 ± 373 *(4)*	>10^6^ *(1)*	>10^6^ *(1)*	*NA^c^*	>>10^6^ *(1)*	*NA^d^*	*NA*
Human peptibody	4ΔhFc	*NA*	*NA*	*NA*	*NA*	>>10^4^ *(1)*	*NA*	>>10^4^ *(1)*	>>10^4^ *(1)*
Peptide^ d^	4Δ	1790 ± 650 *(2)*	4370 ± 2050 *(2)*	*NA*	*NA*	*NA*	*NA*	*NA*	*NA*

a) *n*, number of independent experiments (each performed with 7-8 different concentrations of peptibody in technical duplicates). b) >>, maximum concentration tested that did not reach the IC_50_. c) *NA*, not analyzed. d)*_ac-_*FETLRGDLRILSILRHQNLLKEL*_-CONH2_* (*ac-,* N-terminal acetylated; *-CONH2*, C-terminal amidated).

## Data Availability

All the experimental data of the present study are provided in this article and in the accompanying Supplementary Information. Raw images have been deposited in the San Raffaele Open Research Data repository (https://ordr.hsr.it/research-data/). Request for additional information or resources should be addressed to Flavio Curnis (curnis.flavio@hsr.it).

## Abbreviations

MS: mass spectrometry
LAP: latency-associated peptide
TGFβ: transforming growth factor-β
CgA: Chromogranin A
ESI-MS: Electrospray Ionization Mass Spectrometry
MALDI-TOF: Matrix-Assisted Laser Desorption/Ionization Time of Flight
LC-ESI-MS: Liquid Chromatography coupled to Electrospray Ionization Mass Spectrometry
