# Supplementary material for: An αvβ6/αvβ8-targeting peptibody inhibits integrin-dependent TGFβ activation, enables tumor-selective cytotoxic delivery, and synergizes with PD-L1 immune checkpoint blockade
Source: Theranostics. 2026 Jul 29;16(15):8478–500. doi: 10.7150/thno.131237 (PMC13549134; doi:10.7150/thno.131237)
Supplement: Supplementary file 1 [file thnov16p8478s1.pdf]

Supporting Information

An αvβ6/αvβ8-targeting peptibody inhibits integrin-dependent TGFβ activation, enables tumor-selective cytotoxic delivery, and synergizes with PD-L1 immune checkpoint blockade

Angelo Corti[1,7]*, Anna Maria Gasparri[1], Barbara Colombo[1], Giulia Taiè[1], Elisa Sangiovanni[2], Leopoldo Sitia[1], Valeria Barreca[1], Alice Schirru[2], Alessandro Gori[3], Luc Garrigues[4], Sophie Chabot[4], Annapaola Andolfo[5], Maurilio Ponzoni[6,7], Anna Mondino[2], Arianna Pocaterra[2], Claudio Doglioni[6,7], and Flavio Curnis[1]*

[1]Tumor Biology and Vascular Targeting Unit, Comprehensive Cancer Center, IRCCS San Raffaele Scientific Institute, Milan, Italy;

[2]Lymphocyte Activation Unit, Division of Immunology, IRCCS San Raffaele Scientific Institute, Milan, Italy;

[3]Istituto di Scienze e Tecnologie Chimiche (SCITEC-CNR), National Research Council of Italy, Milan, Italy;

[4]Translational Biology, In vivo Pharmacology and Analytical Chemistry, Evotec, 195, Route D'Espagne, 31100, Toulouse, France;

[5]Proteomics and Metabolomics Facility, Center for Omics Sciences, IRCCS San Raffaele Scientific Institute, Milan, Italy;

[6]Pathology Unit, IRCCS San Raffaele Scientific Institute, Milan, Italy;

[7]Vita-Salute San Raffaele University, Milan, Italy.

Supplemental materials and methods

Surface-Plasmon-Resonance analysis

SPR binding analyses were performed at Evotec Laboratories using two different approaches (*Avidity and affinity set-up,* see below), to assess the avidity and affinity of peptibody-integrin interactions, using a Biacore 8K+ instrument (Cytiva) and single-cycle-kinetics.

Avidity set-up

Recombinant human biotinylated αvβ6 and αvβ8 (Acrobiosystem, cat. IT6-H82E4 and IT8-H82W5, respectively) were reversibly captured on a Series S Sensor Chip CAP using the Biotin CAPture Kit (Cytiva, cat. 28920234), according to the manufacturer's instructions. In this format, the CAP surface captures the Biotin CAPture Reagent (based on avidin), which in turn binds biotinylated integrins, enabling reversible ligand capture and surface renewal at each analysis cycle. Integrins were captured to a level of ~100 RU by injection of 100 nM protein at 5 µl/min for 100 s. Following surface loading, increasing concentrations (range: 0-500 ng/ml; 2-fold serial dilution with 9-points; flow rate: 40 µl/min; association time: 180 s; dissociation time: 1000 s, temperature: 25 °C) of **4Δ**hFc or negative-control hFc (produced in-house) were injected in running buffer consisting of 25 mM HEPES, pH 7.4, 1 mM $MnCl_2$, 1 mM $MgCl_2$, 150 mM NaCl, containing 0.05% Tween-20 (*Binding Buffer-5*). The surface was regenerated with CAP regeneration solution (6 M guanidine hydrochloride in 0.25 M NaOH) for 120 s at 10 µl/min.

Affinity set-up. **4Δ**hFc and control hFc were captured at low surface density on a high-affinity streptavidin-coated sensor chip (Cytiva, cat. 29104992) functionalized with biotinylated CaptureSelect™ Fc multi-species ligand (Thermo Fisher Scientific, cat. 7102852100) to allow capturing of the peptibody via its Fc domain. Capture levels of ~5 RU were used to minimize crowding, rebinding, and secondary avidity effects. After capture of the Fc-fusion proteins (0.5 nM, 60 s, 5 µl/min in *Binding Buffer-5*), various concentrations (range 0-250 nM) of recombinant αvβ6 or αvβ8 integrins were injected as described above. At the end of the study, the sensor chip surface was regenerated with 10 mM glycine pH 2.0 for 120 s at 10 µl/min.

Flow Cytometry

Tumors were excised, weighed, and trimmed to remove macroscopic necrotic areas (if needed). Samples were then mechanically dissociated into small fragments and resuspended in RPMI

containing collagenase A, B, and D (0.5 mg/ml each) and with DNase I (0.1 mg/ml; Roche, cat. 11284932001). Suspensions were gently incubated for 1 h at 37 °C under rotation. After enzymatic digestion, cell suspensions were filtered through a cell strainer (70 µm) and washed in RPMI supplemented with 2% FBS and 2 mM EDTA, pH 8.0. After washing, cells were incubated with ACK (ammonium-chloride-potassium) lysing buffer for 6 min, washed, counted, and aliquoted at $3x10^6$ cells/tube (50 µl/tube). Cells were stained with Zombie Aqua™ Fixable Viability Kit (BioLegend, cat. 423101) according to the manufacturer's instruction. After an additional wash with Ca/Mg-free DPBS, cells were incubated for 15 min with the appropriate panel of fluorochrome-conjugated antibodies (see **Table S4**), diluted in Ca/Mg-free DPBS containing 2% FBS. Antibodies of *Panel 1* were used for surface staining of T-cell populations, whereas those of *Panel 3* were used for macrophage staining. Cells were then washed with Ca/Mg-free DPBS containing 2% FBS, fixed with 2% PFA in Ca/Mg-free DPBS, and stored at 4 °C for 16 h. For cytotoxic effector CD8+ T lymphocyte identification, samples stained with *Panel 1* antibodies were further processed the following day using the FoxP3/Transcription Factor Staining Buffer Set (eBioscience, cat. no. 00-5523-00), according to the manufacturer's instructions and then incubated for 30 min with *Panel 2* antibodies diluted in permeabilization buffer. After washing, cells stained with *Panel 1+2* and *Panel 3* antibodies were analyzed using a CytoFLEX S flow cytometer and FlowJo software. Appropriate fluorescence-minus-one (FMO) controls were used to define gating strategy. Doublets were excluded by gating on forward scatter area versus forward scatter height.

TGFβ ELISA

The effect of **4ΔmFc** on TGFβ levels in TS/A tumors was assessed by ELISA using a commercial kit (DuoSet ELISA, R&D Systems, cat. DY1679-05) specific for active TGFβ1. Tumor tissues were collected from euthanized mice after treatment with **4ΔmFc** or vehicle, weighed, and stored at -80 °C until further usage. To obtain lysates, tumors were homogenized (1 ml/g tissue) in 50 mM Tris-HCl, pH 8.0, 150 mM NaCl, 0.5% (w/v) sodium deoxycholate, 1% (v/v) NP-40, and 0.1% (w/v) SDS, containing a protease inhibitor cocktail (Abcam, cat. ab201119). Homogenates were incubated on ice for 30 min and centrifuged at 20,000xg for 10 min at 4 °C. Supernatants were recovered, aliquoted, and stored at -80 °C for subsequent determination of total protein content (BCA assay) and TGFβ levels. ELISA was performed according to the manufacturer's instructions. For active TGFβ quantification, lysates were diluted 1:10 in PBS containing 0.05% Tween-20 and 1% BSA. For total TGFβ quantification, lysates (10 µl) were diluted in water (190 µl), then acidified with 1 N HCl (40 µl) and incubated

for 10 min at room temperature. Samples were then neutralized with 1.2 N NaOH/0.5 M HEPES (40 µl) and further diluted in PBS containing 0.05% Tween-20 and 1% BSA (final dilution 1:56). TGFβ standard curves were prepared in homogenization buffer and subsequently diluted in PBS containing 0.05% Tween-20 and 1% BSA to minimize matrix effects (0-10 ng/ml final range, 8 serial 1:3 dilution, in duplicate). Samples were transferred to anti-TGFβ-coated wells, incubated 2 h at room temperature, and processed with biotinylated detection antibody (1 h), followed streptavidin-HRP, and TMB substrate according to the manufacturer's instructions.

Tables

Supplementary Table S1. Composition of buffers and binding buffers.

Buffer code	Composition & (Supplier)
DPBS	Dulbecco's phosphate-buffered saline containing calcium and magnesium (EuroClone, cat. EC-B4053L)
DPBS without Ca/Mg	Dulbecco's phosphate buffer saline without calcium and magnesium (EuroClone, cat. EC-B4004L)
PBS	50 mM sodium phosphate buffer pH 7.4, 150 mM sodium chloride
PBS-Sigma	10 mM disodium hydrogen phosphate, 138 mM sodium chloride, 2.7 mM potassium chloride, 1.8 mM potassium dihydrogen phosphate (Merck, cat. P3813-1PAK)
Buffer-1	20 mM Tris-HCl, pH 7.4, 1 mM $MnCl_2$, 1 mM $MgCl_2$, 150 mM NaCl, 0.05% Tween-20
Binding Buffer-1	*Buffer-1* containing 1% BSA and 1% NGS
Buffer-2	20 mM Tris-HCl, pH 7.4, 1 mM $MnCl_2$, 150 mM NaCl, 0.05% Tween-20
Binding Buffer-2	*Buffer-2 containing* 2% BSA
Binding Buffer-3	25 mM HEPES buffer, pH 7.4, 1 mM $MnCl_2$, 1 mM $MgCl_2$, 150 mM NaCl, 0.2% sodium azide, 2% BSA
Binding Buffer-4	25 mM HEPES buffer, pH 7.4, 1 mM magnesium chloride, 1 mM manganese chloride ,150 mM NaCl, 1% BSA
Binding Buffer-5	25 mM HEPES buffer, pH 7.4, 1 mM $MnCl_2$, 1 mM $MgCl_2$, 150 mM NaCl, 0.05% Tween-20
Internalization buffer	DMEM FluoroBrite (Gibco, cat. A1896701) supplemented with 1% glutamine, 1% penicillin/streptomycin, and 10% FBS

Supplementary Table S2. Characterization of peptides by ESI-MS and RP-HPLC.

Peptide sequence [a]	Code	Supplier	Molecular weight (Da) *by ESI-MS*		Purity (%) *by RP-HPLC*
			Expected	Found	
ac-FETLRGDLRILSILRHQNLLKEL-*CONH2*	**4Δ**	In-house	2817.64	2817.65	>95
CFETLRGEERILSILRHQNLLKELQD-*CONH2*	**2a**	In-house	3151.69	3151.69	>95
CFETLRGDLRILSILR**X₁**QNL**X₂**KELQD-*CONH2* [b]	**5a**	In-house/ InnoPep	3120.69	3120.70	>95
ac-HGRGDLGRLKK-*CONH2*	**LAP**-TGFβ3	In-house	1276.74	1276.74	>95
NAVPNLRGDLQVLAQKVART	**A20**FMDV2	ProteoGenix	2162.23	2162.30	>95

a) Single-letter code; ac-, N-terminal acetylated; -*CONH₂*, C-terminal amidated.
b) Triazole-stapled residues (**X₁** and **X₂**, propargylglycine and azidolysine, respectively).

Supplementary Table S3. Plasma half-life of peptide 5a-IRDye and 4ΔmFc-IRDye conjugates [a].

Code	Administration route	Mouse strain	Injected dose (nmol)	Plasma half-life
**5a**-IRDye	Intravenous	BALB/c	1.2	8 min [b]
**4Δ**mFc/IRDye	Intravenous	C57BL/6	0.715	~4 h (early elimination phase)<br>~3 days (late elimination phase)

a) IRDye: IRDye 800 CW (for spectrofluorimetric detection).
b) Reprinted with the permission of Ref. [1].

Supplementary Table S4. Antibodies used for the characterization of tumor infiltrating cells into TS/A tumors by flow cytometry.

Antigen	Antibody clone	Fluorochrome	Supplier	Cat.	Dilution [a)]
Panel 1 *(T-cells)*					
CD45	30-F11	APC-Cy7	BioLegend	103116	1:50
CD4	RM4-5	PE-Cy7	BioLegend	100528	1:200
CD8	53-6.7	PerCP-Cy5.5	BioLegend	100734	1:200
Panel 2					
(Cytotoxic effector CD8+ T cells)					
IFNγ	XMG1.2	FITC	BioLegend	505806	1:50
GrzB	GB11	Pacific Blue	BioLegend	515408	1:50
Panel 3 (Macrophages)					
CD45	30-F11	APC-Cy7	BioLegend	103116	1:50
CD11b	M1/70	PerCP-Cy5.5	BioLegend	101208	1:100
F4/80	BM8	FITC	BioLegend	123120	1:50
CD206	C068C2	PE-Cy7	BioLegend	141720	1:100
CD80	16-10A1	Pacific Blue	BioLegend	104724	1:100

a) Used for staining $3x10^6$ cells resuspended in 50 µl.

Supplemental Figures

```
                                                         ↓
 M   K   H   L   W   F   F   L   L   L   V   A   A   P   R   W   V   L   S   F   E   T   L   R   G
ATG AAG CAC CTG TGG TTC TTC CTG CTG CTG GTG GCC GCT CCA AGA TGG GTT CTG AGC TTC GAG ACC CTG CGG GGA

 D   L   R   I   L   S   I   L   R   H   Q   N   L   L   K   E   L   G   G   G   G   V   P   R   D
GAC TTG AGG ATC CTG AGC ATC CTG CGG CAC CAG AAC CTG CTG AAG GAG CTG GGA GGC GGA GGA GTG CCC AGG GAT

 C   G   C   K   P   C   I   C   T   V   P   E   V   S   S   V   F   I   F   P   P   K   P   K   D
TGT GGT TGT AAG CCT TGC ATA TGT ACA GTC CCA GAA GTA TCA TCT GTC TTC ATC TTC CCC CCA AAG CCC AAG GAT

 V   L   T   I   T   L   T   P   K   V   T   C   V   V   V   D   I   S   K   D   D   P   E   V   Q
GTG CTC ACC ATT ACT CTG ACT CCT AAG GTC ACG TGT GTT GTG GTA GAC ATC AGC AAG GAT GAT CCC GAG GTC CAG

 F   S   W   F   V   D   D   V   E   V   H   T   A   Q   T   Q   P   R   E   E   Q   F   N   S   T
TTC AGC TGG TTT GTA GAT GAT GTG GAG GTG CAC ACA GCT CAG ACG CAA CCC CGG GAG GAG CAG TTC AAC AGC ACT

 F   R   S   V   S   E   L   P   I   M   H   Q   D   W   L   N   G   K   E   F   K   C   R   V   N
TTC CGC TCA GTC AGT GAA CTT CCC ATC ATG CAC CAG GAC TGG CTC AAT GGC AAG GAG TTC AAA TGC AGG GTC AAC

 S   A   A   F   P   A   P   I   E   K   T   I   S   K   T   K   G   R   P   K   A   P   Q   V   Y
AGT GCA GCT TTC CCT GCC CCC ATC GAG AAA ACC ATC TCC AAA ACC AAA GGC AGA CCG AAG GCT CCG CAG GTG TAC

 T   I   P   P   P   K   E   Q   M   A   K   D   K   V   S   L   T   C   M   I   T   D   F   F   P
ACC ATT CCA CCT CCC AAG GAG CAG ATG GCC AAG GAT AAA GTC AGT CTG ACC TGC ATG ATA ACA GAC TTC TTC CCT

 E   D   I   T   V   E   W   Q   W   N   G   Q   P   A   E   N   Y   K   N   T   Q   P   I   M   D
GAA GAC ATT ACT GTG GAG TGG CAG TGG AAT GGG CAG CCA GCG GAG AAC TAC AAG AAC ACT CAG CCC ATC ATG GAC

 T   D   G   S   Y   F   V   Y   S   K   L   N   V   Q   K   S   N   W   E   A   G   N   T   F   T
ACA GAT GGC TCT TAC TTC GTC TAC AGC AAG CTC AAT GTG CAG AAG AGC AAC TGG GAG GCA GGA AAT ACT TTC ACC

 C   S   V   L   H   E   G   L   H   N   H   H   T   E   K   S   L   S   H   S   P   G   K   *
TGC TCT GTG TTA CAT GAG GGC CTG CAC AAC CAC CAT ACT GAG AAG AGC CTC TCC CAC TCT CCT GGT AAA TGA
```

Supplementary Figure S1. Amino acid and cDNA coding sequence of 4ΔmFc.

Upper sequence: **4Δ**mFc protein (amino acids indicated with the single-letter code).

Lower sequence: **4Δ**mFc cDNA (codons optimized for expression in mammalian cells).

*: stop codon.

Boxed sequence: human heavy-chain signal peptide for peptibody secretion into the culture medium.

Arrow: expected cleavage site of the fusion protein.

Bold: CgA-derived peptide **4Δ**.

Gray box: four-glycine spacer.

Underlined sequence: murine IgG1-Fc region, consisting of hinge plus CH2 and CH3 domains.

```
                                                                                          ↓
 M   K   H   L   W   F   F   L   L   L   V   A   A   P   R   W   V   L   S   F   E   T   L   R   G
ATG AAG CAC CTG TGG TTC TTC CTG CTG CTG GTG GCT GCC CCT AGG TGG GTG CTG AGC TTT GAG ACC CTG AGG GGC

 D   L   R   I   L   S   I   L   R   H   Q   N   L   L   K   E   L   G   G   G   G   E   P   K   S
GAT CTG AGG ATC CTG TCC ATC CTG CGG CAC CAG AAC CTG CTG AAG GAG CTG GGC GGC GGC GGA GAA CCA AAG AGC

 Q   D   K   T   H   T   C   P   P   C   P   A   P   E   L   L   G   G   P   S   V   F   L   F   P
CAG GAT AAG ACC CAC ACC TGT CCT CCT TGC CCT GCC CCT GAG CTG CTG GGA GGA CCT TCC GTG TTT CTG TTC CCT

 P   K   P   K   D   T   L   M   I   S   R   T   P   E   V   T   C   V   V   V   D   V   S   H   E
CCT AAG CCC AAG GAT ACC CTG ATG ATC AGC CGG ACC CCT GAG GTG ACC TGT GTG GTG GTG GAT GTG AGC CAC GAG

 D   P   E   V   K   F   N   W   Y   V   D   G   V   E   V   H   N   A   K   T   K   P   R   E   E
GAC CCT GAG GTG AAG TTC AAC TGG TAT GTG GAT GGC GTG GAG GTG CAC AAT GCT AAG ACC AAG CCT CGG GAG GAG

 Q   Y   N   S   T   Y   R   V   V   S   V   L   T   V   L   H   Q   D   W   L   N   G   K   E   Y
CAG TAC AAC AGC ACC TAT AGG GTG GTG AGC GTG CTG ACC GTG CTG CAC CAG GAC TGG CTG AAC GGC AAG GAG TAT

 K   C   K   V   S   N   K   A   L   P   A   P   I   E   K   T   I   S   K   A   K   G   Q   P   R
AAG TGT AAG GTG AGC AAC AAG GCT CTG CCT GCT CCC ATC GAG AAG ACC ATC AGC AAG GCT AAG GGC CAG CCT AGG

 E   P   Q   V   Y   T   L   P   P   S   R   D   E   L   T   K   N   Q   V   S   L   T   C   L   V
GAG CCT CAG GTG TAC ACC CTG CCC CCC AGC AGA GAC GAG CTG ACC AAG AAC CAG GTG TCC CTG ACC TGC CTG GTG

 K   G   F   Y   P   S   D   I   A   V   E   W   E   S   N   G   Q   P   E   N   N   Y   K   T   T
AAG GGC TTT TAC CCC AGC GAC ATC GCC GTG GAG TGG GAG AGC AAT GGC CAG CCC GAG AAC AAT TAT AAG ACC ACC

 P   P   V   L   D   S   D   G   S   F   F   L   Y   S   K   L   T   V   D   K   S   R   W   Q   Q
CCC CCC GTG CTG GAC TCC GAC GGA TCT TTC TTC CTG TAT TCC AAG CTG ACC GTG GAC AAG AGC CGG TGG CAG CAG

 G   N   V   F   S   C   S   V   M   H   E   A   L   H   N   H   Y   T   Q   K   S   L   S   L   S
GGC AAT GTG TTT AGC TGC AGC GTG ATG CAC GAG GCC CTG CAC AAC CAC TAT ACC CAG AAG AGC CTG AGC CTG TCC

 P   G   K   *
CCT GGC AAG TGA
```

Supplementary Figure S2. Amino acid and cDNA coding sequence of 4ΔhFc.

Upper sequence: **4Δ**hFc protein (amino acids indicated with the single-letter code).

Lower sequence: **4Δ**hFc cDNA (codons optimized for expression in mammalian cells).

*: stop codon.

Boxed sequence: human heavy-chain signal peptide for peptibody secretion into the culture medium.

Arrow: expected cleavage site of the fusion protein.

Bold: CgA-derived peptide **4Δ**.

Gray box: four-glycine spacer.

Underlined sequence: human IgG1-Fc region, consisting of a hinge with Cys226Gln substitution (*highlighted in yellow*) plus CH2 and CH3 domains.

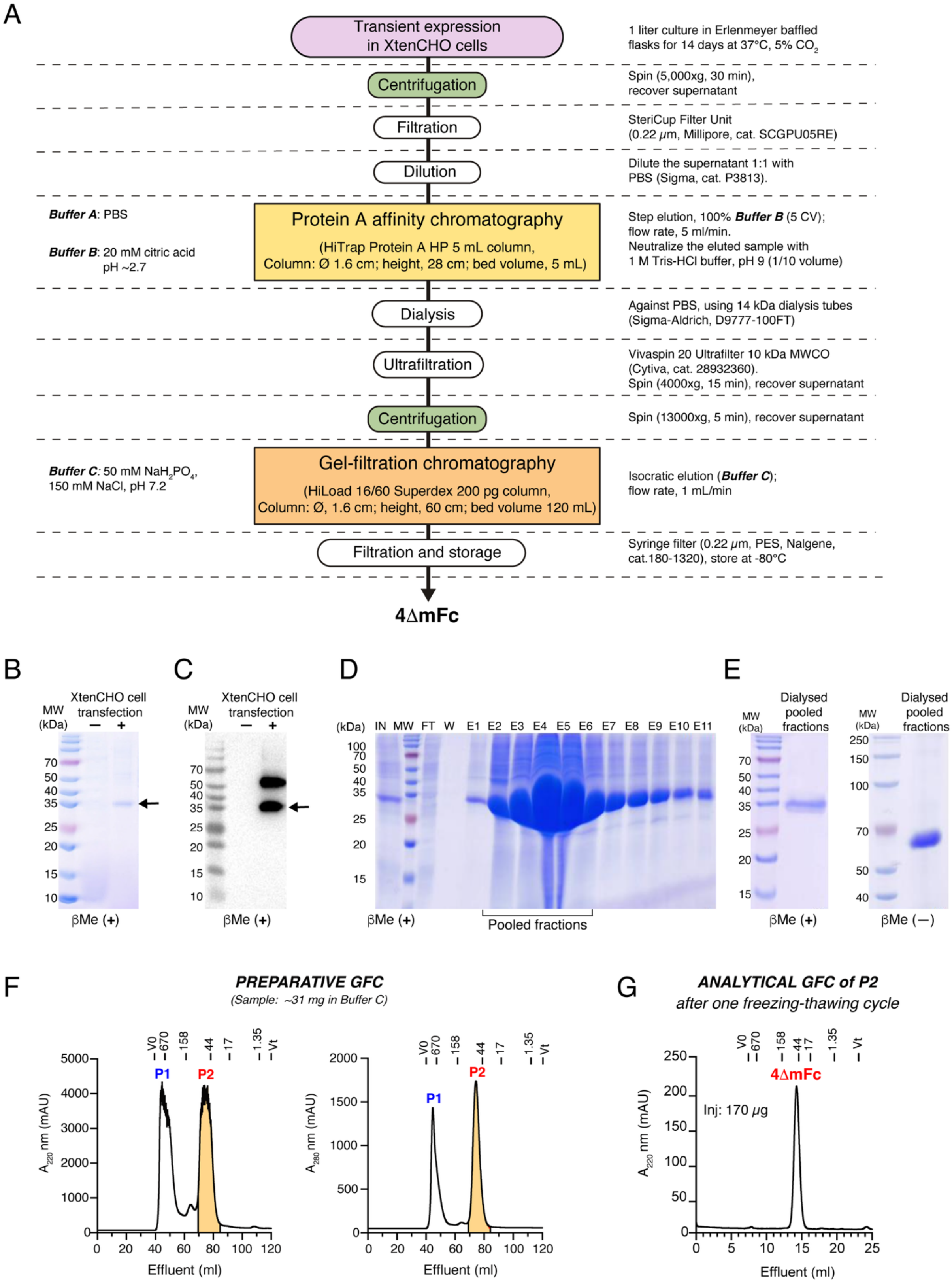

Supplementary Figure S3. Production and purification of 4ΔmFc.

A) Schematic workflow of **4ΔmFc** purification. The purification steps and materials used are indicated. The entire procedure requires approximately 18 days (*see Materials and Methods* section in the main text).

B) SDS-PAGE and **C**) Western blot analysis of culture supernatants from XtenCHO cells transfected with pTXs1/**4ΔmFc** plasmid (+) or left untreated (–) after 14 days at 37 °C, 5% CO_2. Western blot was performed using an HRP-conjugated anti-Fc polyclonal antibody. The arrow indicates a ~35 kDa band consistent with the **4ΔmFc** product.

D) SDS-PAGE analysis under reducing conditions (βMe +) of Protein A affinity chromatography fractions. *IN*, input; *MW*, molecular weight marker; *FT*, flow-through; W, wash fraction; E1-E22, eluted fractions. The bar indicates the pooled fractions that were subsequently dialyzed.

E) SDS-PAGE analysis of the Protein A affinity chromatography product after dialysis, under both reducing (βMe +) and non-reducing (βMe –) conditions.

F) Preparative gel filtration chromatography (GFC) of the Protein A product (31 mg) on a HiLoad 16/60 Superdex 200 column, monitored at A_{220} nm and A_{280} nm. Peak 1 (*P1*) corresponds to high molecular-weight aggregates. Peak 2 (*P2*, yellow area), which contained the material of the expected molecular weight, was pooled, sterile-filtered, and kept at –80 °C. The final purified product was called **4ΔmFc**. Vo, *void volume*; Vt, *total volume*; and the elution volumes of molecular-weight standards (670, 158, 44, 17, and 1.35 kDa) are indicated.

G) Analytical gel filtration chromatography of **4ΔmFc** (*P2*) after one freeze-thaw cycle, analyzed on a Superdex 200 10/300 GL column.

Experiments described in *Panels B-E* were carried out by ProteoGenix.

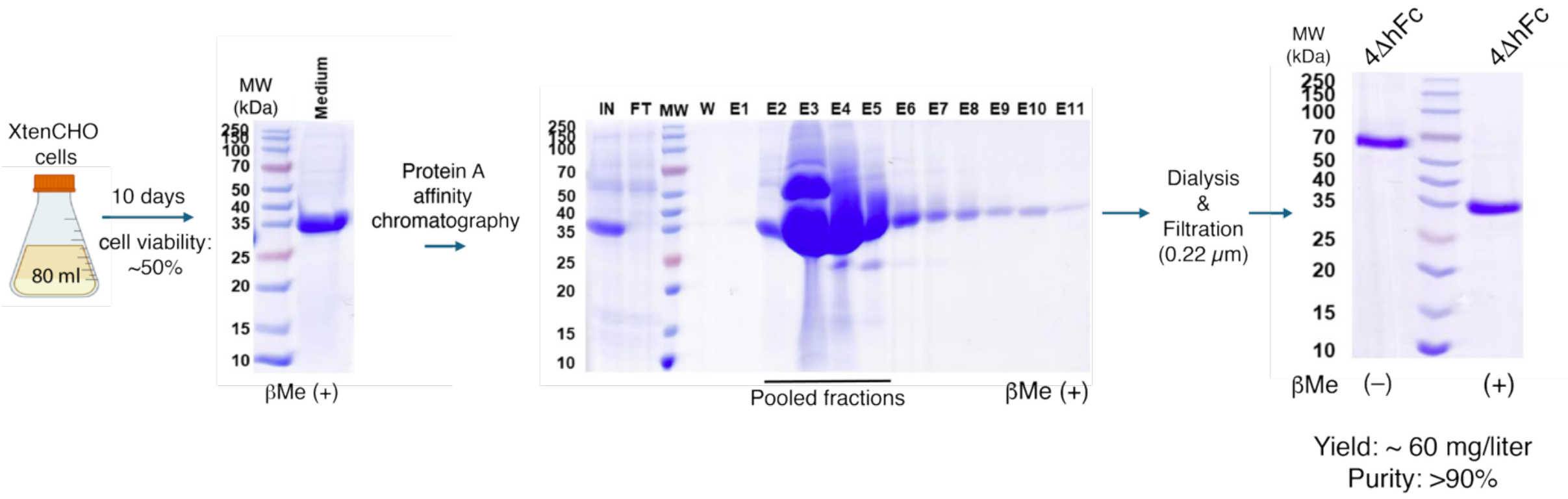

Supplementary Figure S4. Expression, purification, and functional characterization of the peptibody 4ΔhFc.

A) Schematic workflow for production and purification of **4ΔhFc** as monitored by SDS-PAGE analysis. The peptibody was transiently expressed in mammalian XtenCHO cells (*left gel*) and purified from the culture medium by Protein A affinity chromatography (*central gel*). Fractions E2-E5 were pooled, dialyzed, sterile-filtered, and analyzed under non-reducing (βMe –) and reducing (βMe +) conditions (*right gel*). These experiments were carried out by ProteoGenix.

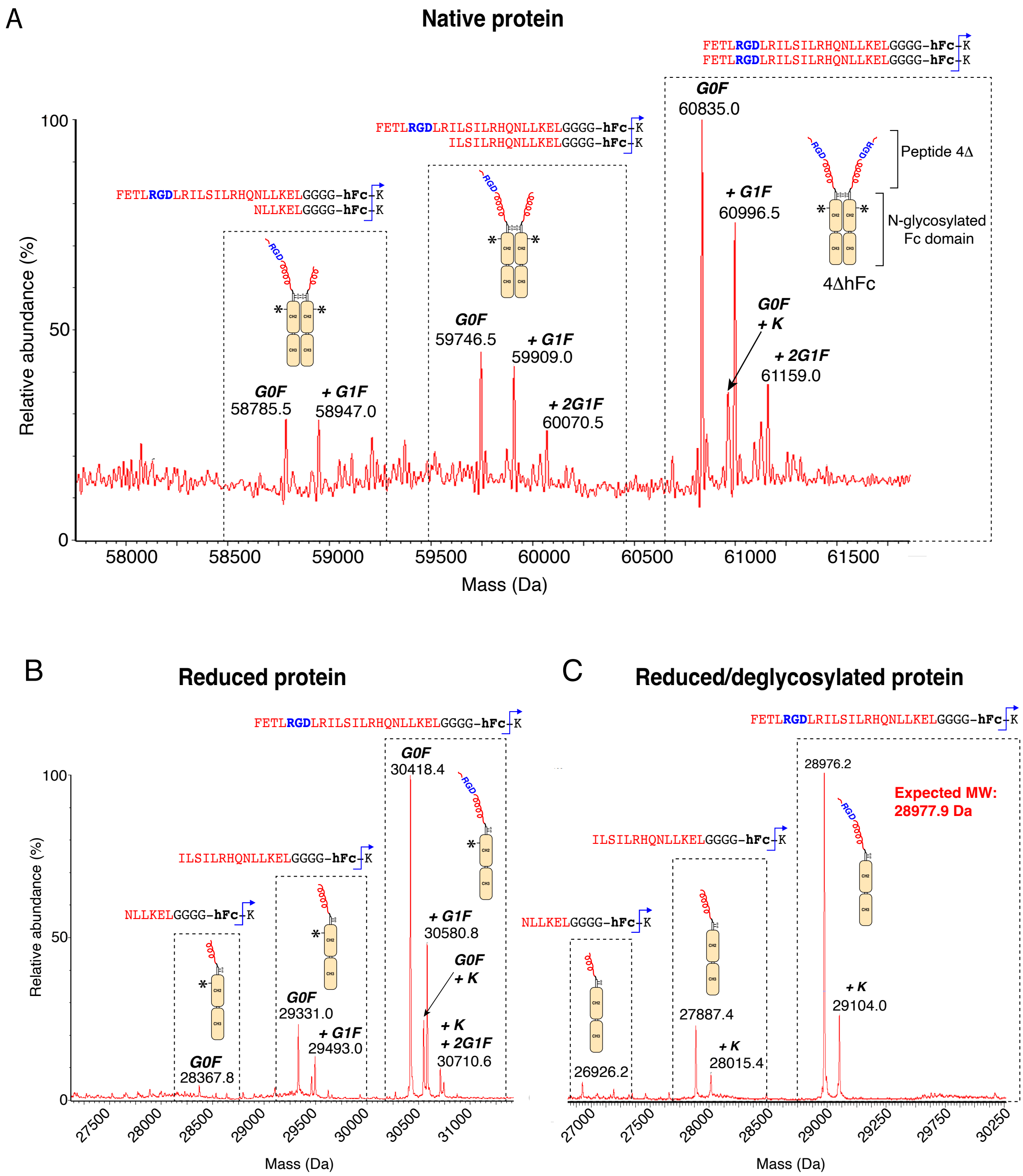

Supplementary Figure S5. LC-ESI-MS characterization of the human peptibody 4ΔhFc. **A)** Deconvoluted mass spectrum of the native (non-reduced) **4Δ**hFc showing a major species at 60835.0 Da, consistent with the full-length disulfide-linked dimer bearing two G0F N-glycans (one per Fc chain) and predominantly lacking the C-terminal lysine residues. Minor species at 59746.5 Da and 58785.5 Da are consistent with heterodimers composed of one full-length glycosylated chain and one N-terminally truncated chain lacking FETLRGDLR (59746.5 Da) or FETLRGDLRILSILRHQ (58785.5 Da). For both the full-length and truncated forms, additional more galactosylated glycoforms were detected (indicated as +G1F and +2G1F), as well as variants retaining the C-terminal lysine (G0F +K). The expected N-terminal

sequences of the most abundant species and their schematic representations are reported; *blue arrows* indicate C-terminal lysine clipping.

B) Deconvoluted mass spectrum of the reduced **4Δ**hFc. The major peak at 30418.4 Da corresponds to the full-length monomeric chain carrying a G0F glycoform and predominantly lacking the C-terminal lysine; less abundant peaks corresponding to G1F/2G1F glycoforms, and/or +K variants are indicated.

C) Deconvoluted mass spectrum of the reduced, PNGase F-treated (deglycosylated) **4Δ**hFc. The major peak had a mass of 28976.2 Da, corresponding to a loss of 1442 Da, in good agreement with the removal of a G0F-glycoform (expected 1463.5 Da) and with the expected molecular weight (28977.9 Da) of the intact peptibody without the C-terminal lysine.

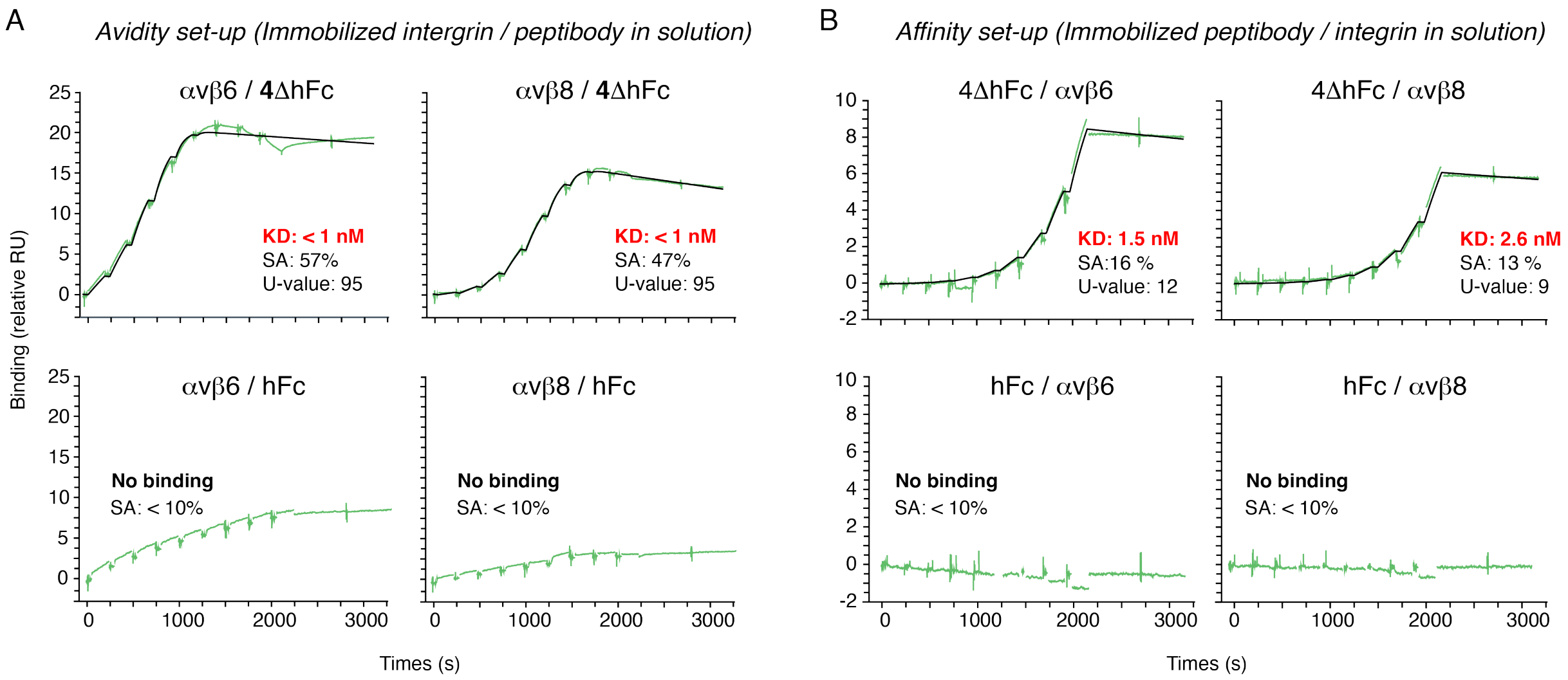

Supplementary Figure S6. SPR analysis of 4ΔhFc and hFc binding to αvβ6 and αvβ8.

A) Avidity set-up sensorgrams obtained by injecting increasing concentrations of **4Δ**hFc or control hFc (*upper* and *lower panels, respectively*) over biotinylated αvβ6 (*left*) or αvβ8 (*right*) captured on the sensor chip.

B) Affinity set-up sensorgrams obtained by injecting increasing concentrations of αvβ6 (*left*) or αvβ8 (*right*) over immobilized **4Δ**hFc (*upper panels*) or control hFc (*lower panels*) captured on the sensor chip via their Fc domain.

The data were collected on a Biacore 8K+ instrument using Single-Cycle Kinetics and analyzed with a 1:1 kinetic binding model. Green lines represent experimental data, and black lines correspond to the fitted curves. The calculated equilibrium dissociation constant (*KD*), surface activity (*SA*), and units of immobilization (*U-value*) are reported in each panel. SA values <10% indicate no binding, while U-values >25 indicate poor fit quality.

Note that in the avidity set-up (**A**), KD values should be considered apparent only (KD_{app}), because the dissociation rate for both integrins was outside the instrument detection limits; furthermore, the high U-values indicate poor uniqueness of the fitted kinetic parameters. Notably, control hFc showed modest binding for the immobilized αvβ6 in the avidity set-up with SA<10% (**A**, *lower left*), while it showed no binding to αvβ6 integrin in the affinity set-up (**B**, *lower left*), therefore, this binding was attributed to non-specific interaction.

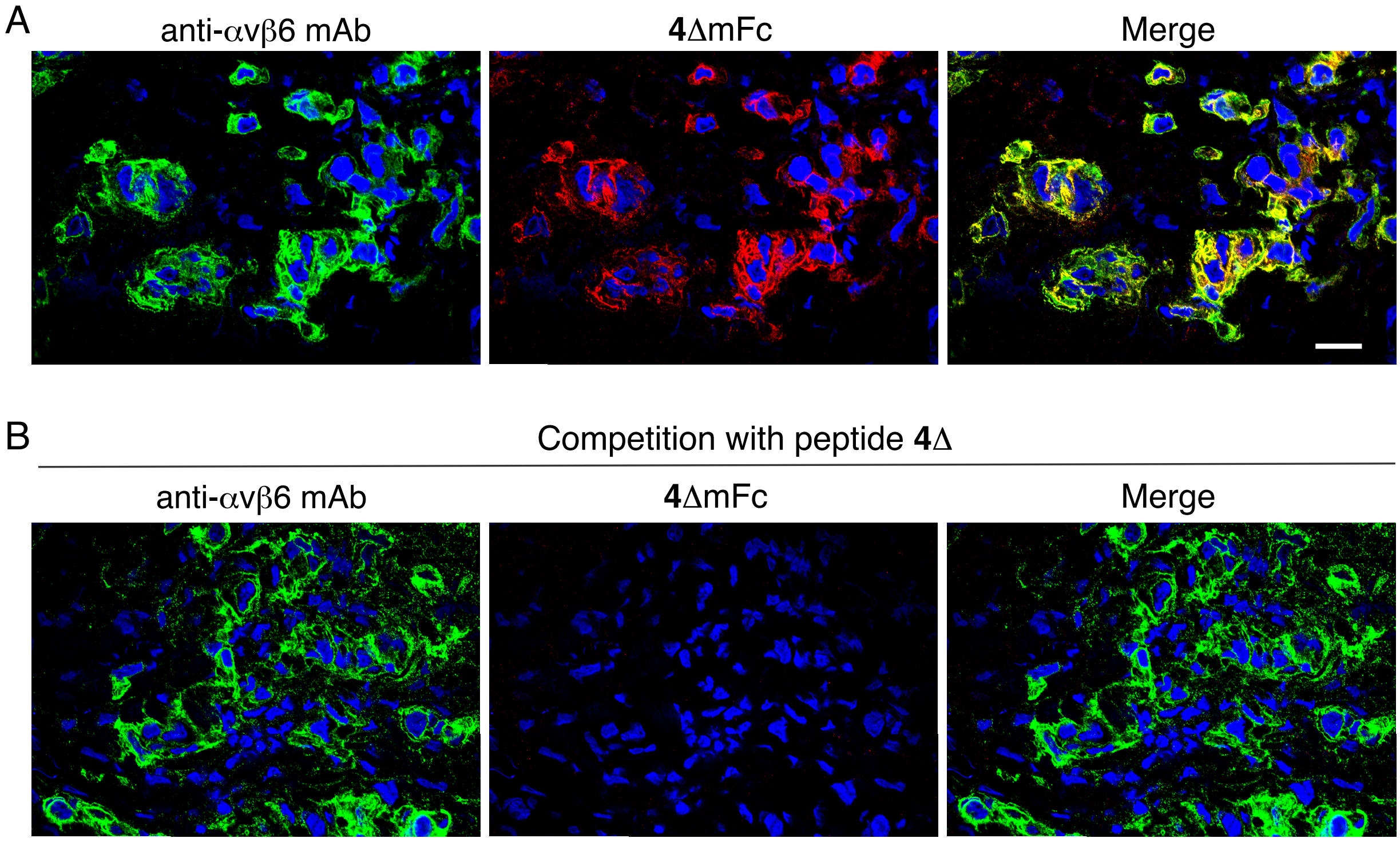

Supplementary Figure S7. Murine peptibody binds to colon cancer cells in human tissue, and an excess of free peptide 4Δ completely abrogates its binding.

Co-staining of human colon carcinoma tissue sections with a rabbit anti-αvβ6 mAb (*green*) and the peptibody **4Δ**mFc (*red*) in the absence (**A**) or presence (**B**) of an excess of free peptide **4Δ** as detected by confocal immunofluorescence microscopy using an Olympus FluoVIEW FV3000RS system. Peptide **4Δ** was added at a 500-fold excess (~5 µM) over **4Δ**mFc during both the blocking and binding steps. Nuclei were counterstained with DAPI (*blue*). Representative confocal photomicrographs of individual channels and corresponding merged images are shown. Scale bar, 20 µm.

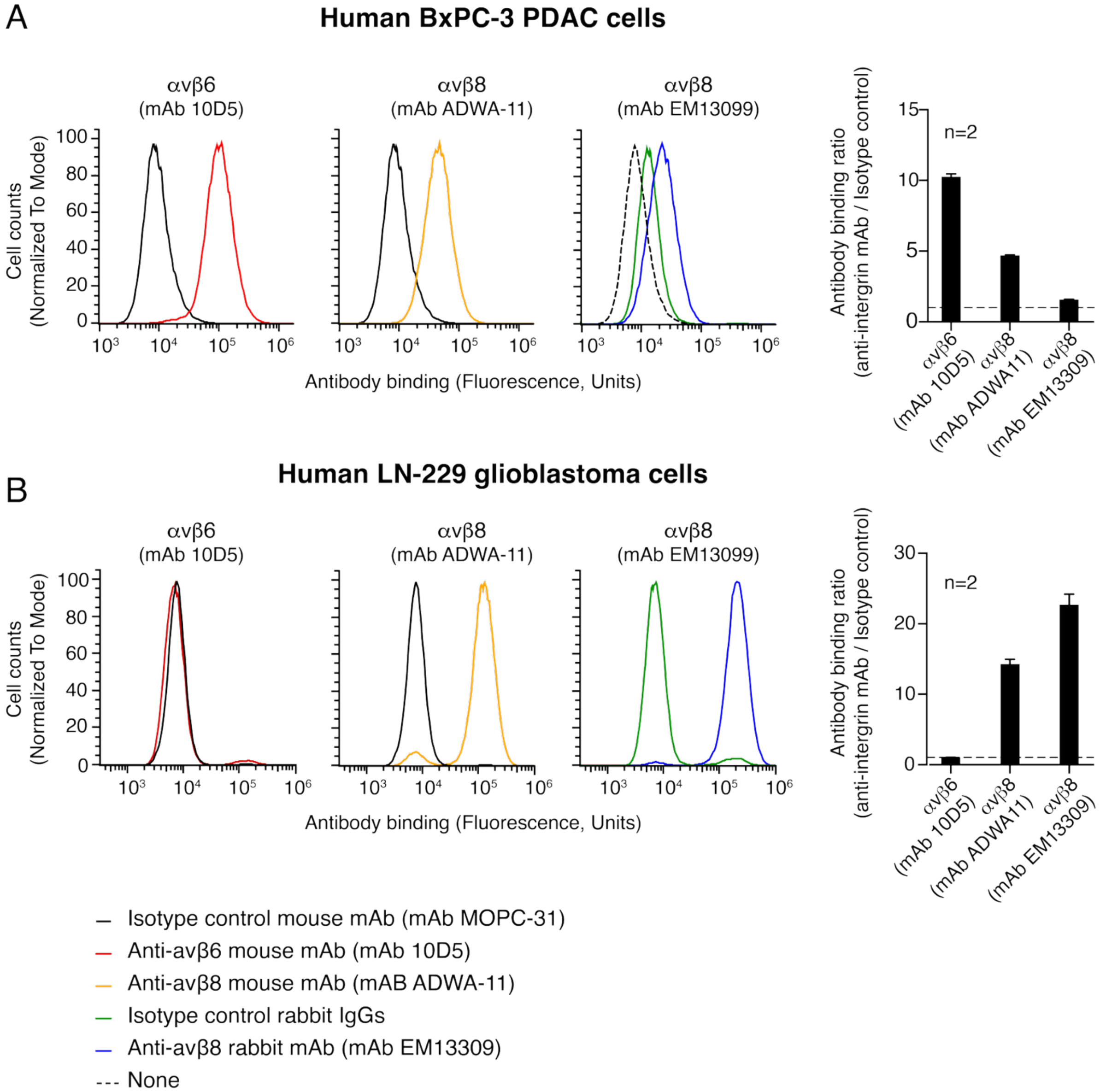

Supplementary Figure S8. Expression of αvβ8 and αvβ6 by LN-229 and BxPC-3 cells, as assessed by flow cytometry.

Flow cytometry was carried out using the indicated primary antibodies, followed by Alexa Fluor 488-conjugated goat anti-rabbit or goat anti-mouse IgG polyclonal secondary antibodies. These experiments were performed using a new batch of BxPC-3 cells obtained from ATCC, which expressed the αvβ8 integrin, whereas previous studies reported little or no expression of this integrin [1].

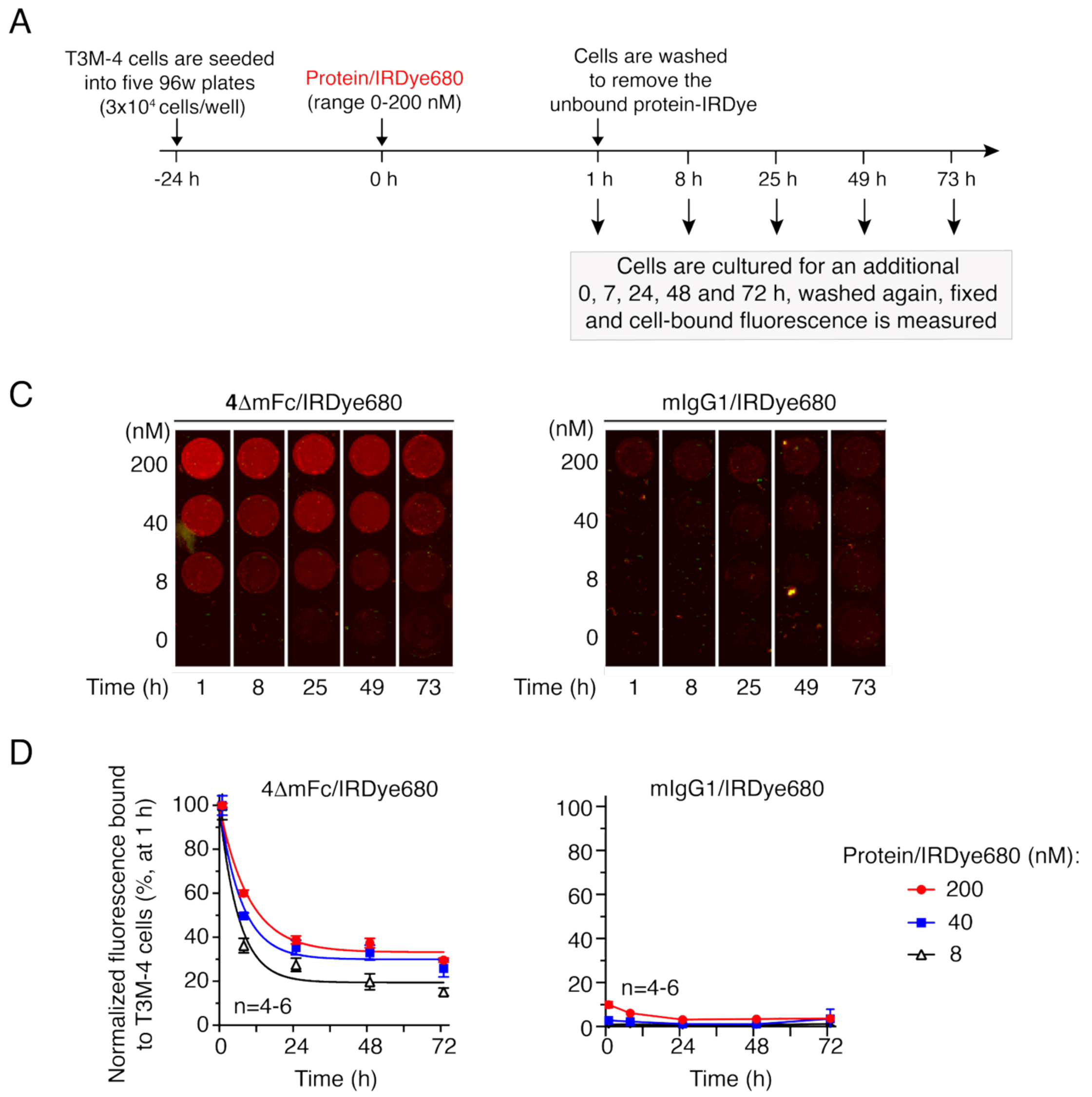

Supplementary Figure S9. Pulse-chase analysis of 4ΔmFc/IRDye680 and mIgG1/IRDye680 after washout. A) Scheme of the experiment. T3M-4 cells were seeded in five 96-well plates and incubated for 1 h with 8, 40, or 200 nM of IRDye680-labeled **4ΔmFc** or mIgG1 control. Cells were then washed to remove unbound fluorescently labeled proteins and further cultured for 0, 7, 24, 48, or 72 h before fixation and fluorescence analysis.

B) Representative fluorescence images of T3M-4 cells at the indicated time points after washout, incubated with **4ΔmFc**/IRDye680 (*left panel*) or mIgG1/IRDye680 (*right panel*).

C) Quantification of residual cell-associated fluorescence in T3M-4 cells. For the peptibody, the fluorescence signal measured after washout (0 h) was set to 100%, and values at later time points were expressed relative to the corresponding initial signal, while the fluorescence signal of the mIgG$_1$ control was expressed as a percentage of the corresponding **4ΔmFc** signal. Dots represent mean ± SE of n=4-6 wells (in some cases, error bars are smaller than the symbols).

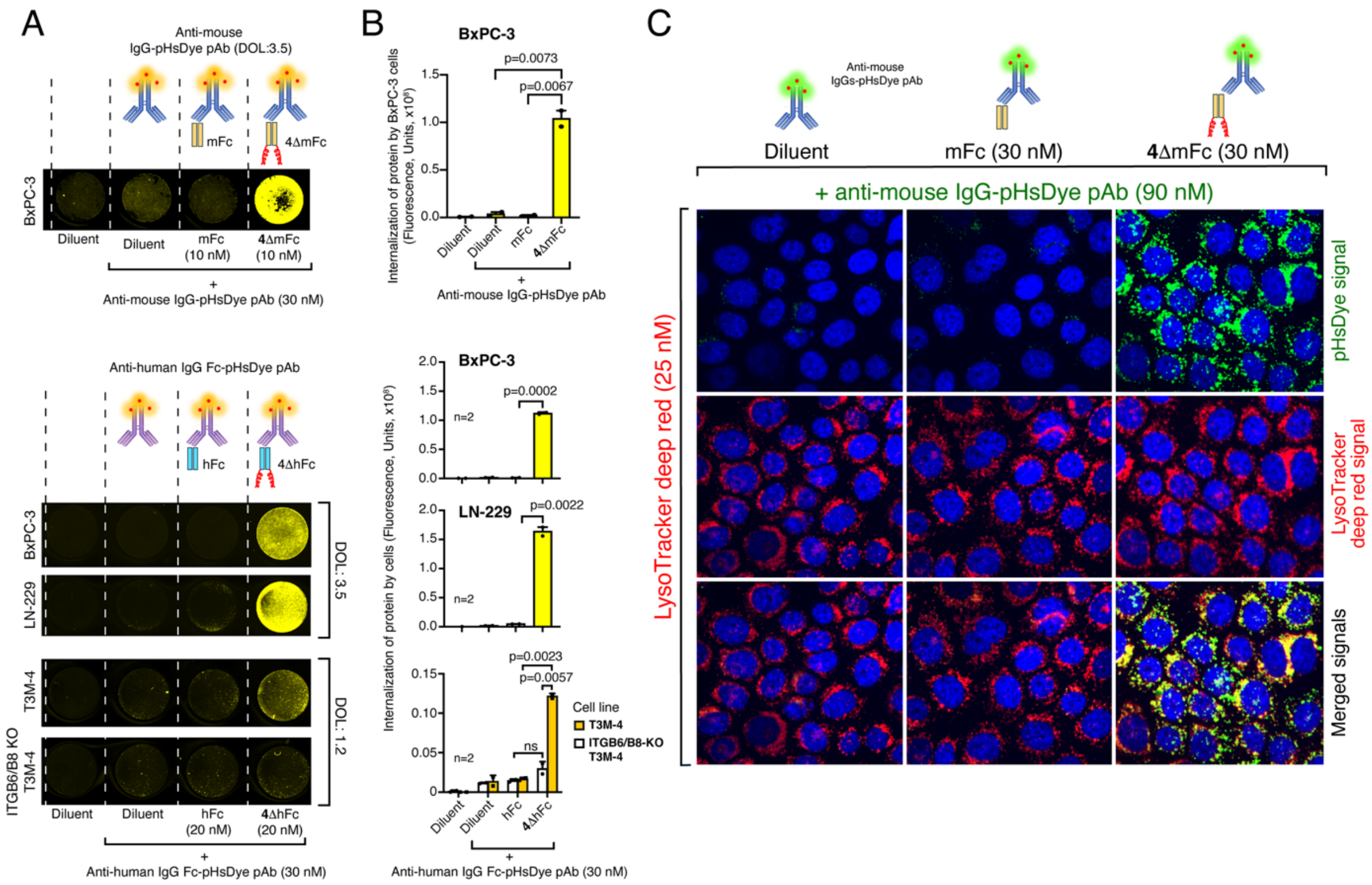

Supplementary Figure S10. Murine and human peptibodies enable efficient internalization of antibody conjugates.

A) Schematic representation of the experimental setup and representative microtiter-well images showing the internalization of murine (*upper panel*) and human peptibodies (*lower panels*). The indicated secondary polyclonal antibodies (pAb) labeled with a pH-sensitive dye (pHsDye) were pre-complexed with either the indicated peptibodies, a control Fc fragment, or diluent alone and then added to the indicated cells. After 20 h of incubation, cells were washed, and the plate fluorescence was acquired with a Sapphire imager scanner. Representative microtiter-well images are shown; yellow fluorescence corresponds to the intracellular signal. The degree of labelling (DOL; number of fluorescent molecules per antibody) used in each experiment is reported in each panel.

B) Quantification of internalized fluorescence by the indicated cells. Bars, mean ± SE of technical replicates (n=2 wells). *P* values, calculated by two-tailed unpaired t-test, are indicated in the plots.

C) 4ΔmFc-pHsDye is efficiently internalized into the lysosomal/endosomal compartments. **4Δ**mFc-pHsDye, control mFc, or diluent were pre-incubated for 10 min with an anti-mouse IgG polyclonal antibody conjugated to a pH-sensitive dye (anti-mouse IgG-pHsDye, DOL: 3.5) at the indicated concentrations and then added to BxPC-3 cells. Cells were incubated overnight at 37 °C, 5% CO_2, washed, and stained with LysoTracker™ Deep Red to label

lysosomes/endosomes and with Hoechst 33258 to stain the nuclei. After a final wash, the cell-associated fluorescence was acquired using a wide-field confocal microscope equipped with a 40× objective. Single-channel and merged images are shown for LysoTracker™ Deep Red (*red*) and protein-pHsDye conjugates (*green*). Hoechst 33258 fluorescence (*blue*) is displayed in all images. Note that the pHsDye signal is displayed in green to distinguish it from LysoTracker Deep Red, whereas in *Panel A,* it is pseudocolored in yellow to enhance contrast on a dark background.

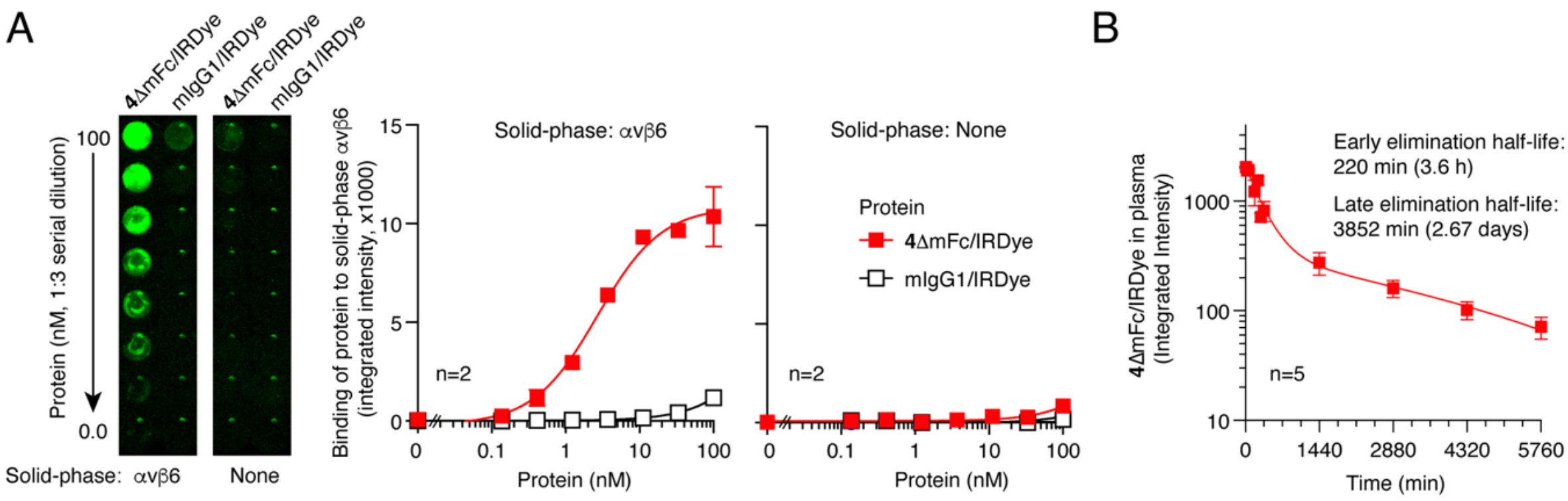

Supplementary Figure S11. Pharmacokinetics of 4ΔmFc/IRDye in mice

A) Binding of **4Δ**mFc/IRDye and mIgG/IRDye to microtiter plates coated with or without human recombinant αvβ6 (2 µg/ml). The assay was carried out as described previously [1]. Bound fluorescence was quantified using an infrared scanner (Odyssey CLx, LI-COR). Representative images of the scanned microplates and binding quantification are shown. Mean ± SE of n = 2 wells.

B) Plasma pharmacokinetics of **4Δ**mFc/IRDye in mice. C57BL/6 mice (n = 5) were injected i.v. with **4Δ**mFc/IRDye (50 µg/mouse). Blood samples were collected in heparinized tubes at various time points. Plasma aliquots (20 µl) were diluted with PBS to 50 µL, and transferred to 96-well microtiter plates. Fluorescence intensity was quantified with an Odyssey scanner. Mean ± SE of 5 mice. The plasma half-life of **4Δ**mFc/IRDye was calculated using a two-phase exponential decay equation (GraphPad Prism software).

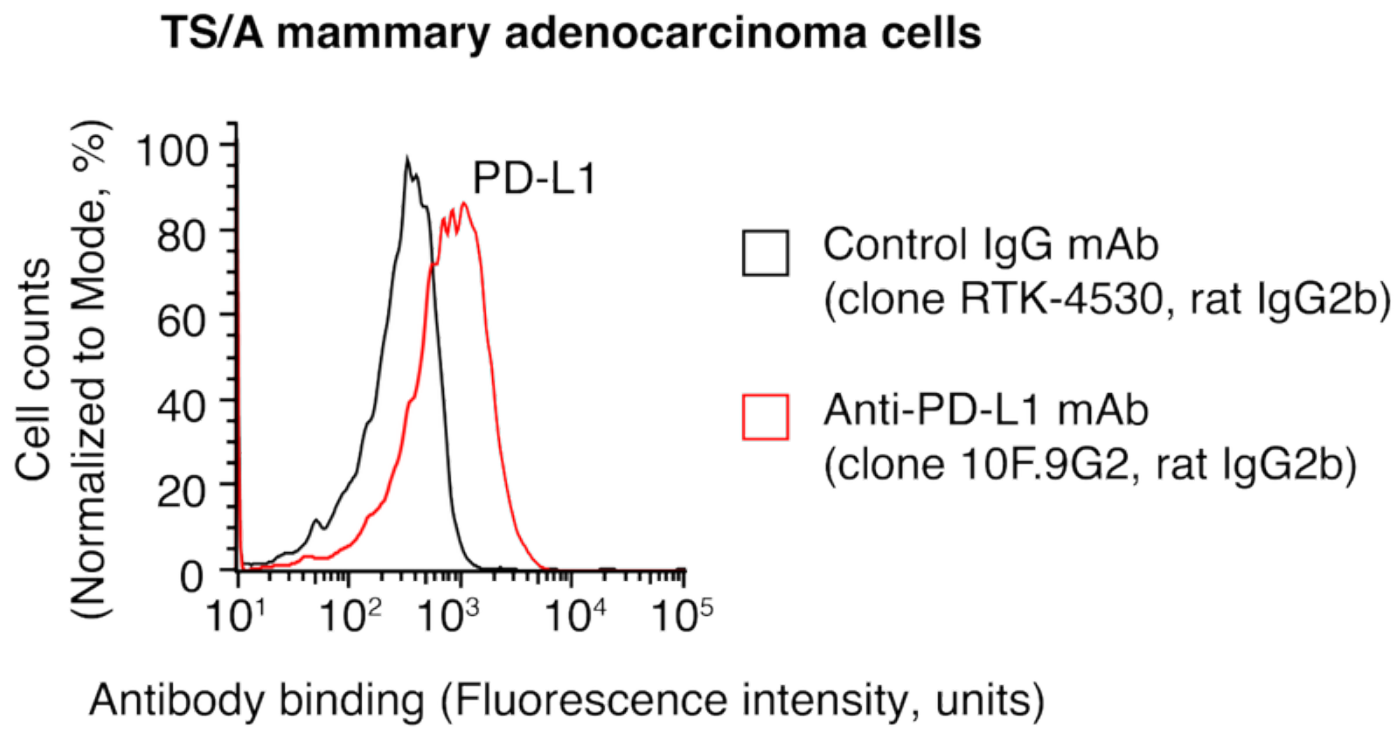

Supplementary Figure S12. PD-L1 expression by TS/A cells.

FACS analysis of cells was performed using the anti-PD-L1 mAb 10F.9G2 (5 µg/ml) followed by a goat anti-rat Alexa Fluor 488-labeled secondary antibody (5 µg/ml). The binding of an isotype control antibody (clone RTK-4530) is also shown.

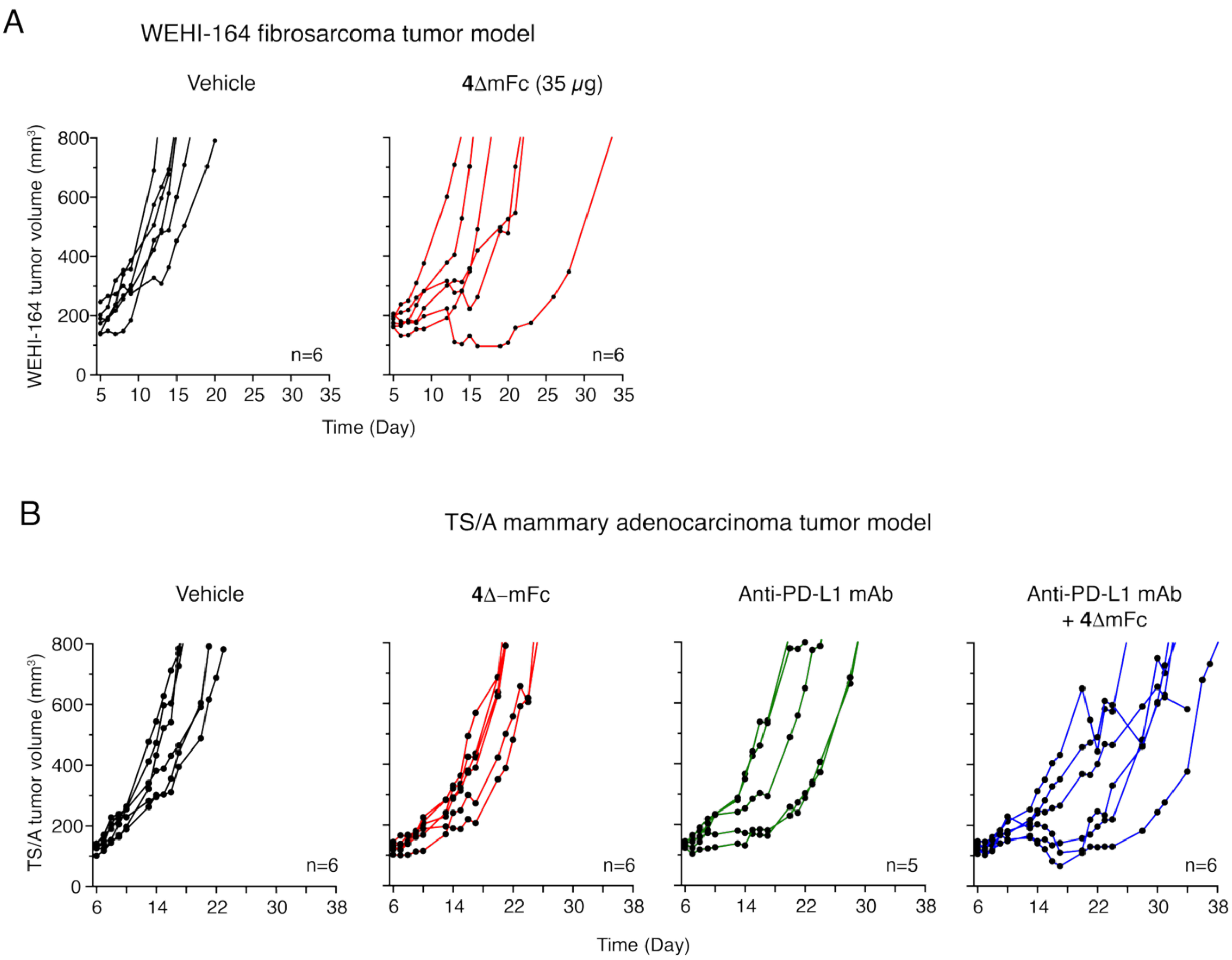

Supplementary Figure S13. Pharmacological effects of 4ΔmFc alone or in combination with immunotherapy in tumor-bearing mice.

Individual tumor growth curves of subcutaneous WEHI-164 (A) and TS/A (B) tumors treated as reported in Figures 8 and 9 of the main text. Mice were euthanized when tumor volume exceeded 800-900 mm³, or earlier if marked tumor ulceration or other signs of distress were observed.

Supplementary References

1. Monieri M, Rainone P, Sacchi A, Gori A, Gasparri AM, Coliva A, et al. A stapled chromogranin A-derived peptide homes in on tumors that express αvβ6 or αvβ8 integrins. Int J Biol Sci. 2023; 19: 156-66.
